# Mineral Nutrition and the Risk of Chronic Diseases: A Mendelian Randomization Study

**DOI:** 10.3390/nu11020378

**Published:** 2019-02-12

**Authors:** Wen-Wen Cheng, Qiang Zhu, Hong-Yu Zhang

**Affiliations:** Hubei Key Laboratory of Agricultural Bioinformatics, College of Informatics, Huazhong Agricultural University, Wuhan 4300700, China; chengww@webmail.hzau.edu.cn (W.-W.C.); zhy630@mail.hzau.edu.cn (H.-Y.Z.)

**Keywords:** calcium, magnesium, iron, copper, zinc, Mendelian randomization, chronic diseases

## Abstract

We applied Mendelian randomization analyses to investigate the potential causality between blood minerals (calcium, magnesium, iron, copper, and zinc) and osteoporosis (OP), gout, rheumatoid arthritis (RA), type 2 diabetes (T2D), Alzheimer’s disease (AD), bipolar disorder (BD), schizophrenia, Parkinson’s disease and major depressive disorder. Single nucleotide polymorphisms (SNPs) that are independent (*r*^2^ < 0.01) and are strongly related to minerals (*p* < 5 × 10^−8^) are selected as instrumental variables. Each standard deviation increase in magnesium (0.16 mmol/L) is associated with an 8.94-fold increase in the risk of RA (*p* = 0.044) and an 8.78-fold increase in BD (*p* = 0.040) but a 0.10 g/cm^2^ increase in bone density related to OP (*p* = 0.014). Each per-unit increase in copper is associated with a 0.87-fold increase in the risk of AD (*p* = 0.050) and BD (*p* = 0.010). In addition, there is suggestive evidence that calcium is positively correlated (OR = 1.36, *p* = 0.030) and iron is negatively correlated with T2D risk (OR = 0.89, *p* = 0.010); both magnesium (OR = 0.26, *p* = 0.013) and iron (OR = 0.71, *p* = 0.047) are negatively correlated with gout risk. In the sensitivity analysis, causal estimation is not affected by pleiotropy. This study supports the long-standing hypothesis that magnesium supplementation can increase RA and BD risks and decrease OP risk and that copper intake can reduce AD and BD risks. This study will be helpful to address some controversial debates on the relationships between minerals and chronic diseases.

## 1. Introduction

Despite the great advances that have been made in the prevention and treatment of chronic diseases, such as osteoporosis (OP), gout, rheumatoid arthritis (RA), type 2 diabetes (T2D), Alzheimer’s disease (AD), bipolar disorder (BD), schizophrenia (SCZ), Parkinson’s disease (PD), and major depressive disorder (MDD), the etiology and mechanisms of many diseases are not fully understood [1,2]. A better understanding of causal mechanisms will enable the prevention of chronic diseases, direct the launch of proper clinical trials, and provide targets for effective lifestyle and pharmacological interventions. Understanding which clinical risk factors should be targeted to reduce the risk of chronic diseases is important. Some observational studies have shown that there is a close relationship between minerals and human diseases. For instance, moderate calcium (Ca) supplementation helps reduce T2D risk [3]; higher magnesium (Mg) is negatively associated with OP and AD [4,5]; iron (Fe) and Zn are negatively correlated with MDD [6]; and Mg, copper (Cu) and zinc (Zn) are positively correlated with RA [7] (Supplementary Table S1). However, these observational findings are difficult to interpret because these results are also affected by a variety of confounding factors, such as occupational and environmental exposures, diet, and supplement use [8]. 

At present, with the extensive publication of data such as DNA methylation, copy number variants, quantitative trait locus (QTL), and genome-wide association study (GWAS), causal inference using exposure-related genetic variation as an instrumental variable (IV), i.e., Mendelian randomization (MR), provides a way forward [9]. The MR method follow the purpose that the parental allele is randomly assigned to the offspring, so it is less likely to be disturbed by confounding factors; because the genotype is not affected by the disease, it also avoids reverse causal bias [10]. MR has found utility across a wide range of domains. On the one hand, MR promotes the development of pharmaceutical agents (i.e., drug target validation, drug target repurposing, and side effect identification) [10]. On the other hand, MR successfully estimated the unbiased association between exposure factors and disease risk. For example, an MR study indicates that obesity may be a risk factor for gout [11], and education levels are negatively related to AD risk [12]. Accordingly, Cu supplementation can effectively reduce the incidence and mortality of coronary heart disease [13], and elevated Ca levels increase the risk of migraine [14]. However, to our knowledge, the effects of minerals in chronic diseases have not been evaluated on a large scale with the use of MR. A two-sample analysis strategy may be employed in which evidence for the correlation between exposure factors (minerals) and outcome factors (chronic diseases) is taken from different samples [15].

This study uses the relationship between minerals and chronic diseases in traditional epidemiological investigations as a background to validate long-standing epidemiological assumptions and to help resolve some controversial debates. We selected data for MR analysis following two criteria: exposure factors had at least two significant (*p* < 5 × 10^−8^) single nucleotide polymorphisms (SNPs) in a GWAS study and summary-level data for the SNP-outcome associations were obtained from the published shared data; there was a traditional epidemiological survey as a background for the link between exposure and outcome factors. In the end, we performed a two-sample MR analysis to evaluate the causality between five minerals (Ca, Mg, Fe, Cu, and Zn) and nine chronic diseases (OP, gout, RA, T2D, AD, BD, SCZ, PD, and MDD) on large-scale GWAS summary data (Supplementary Table S1). We found that each standard deviation (SD) supplementation in Mg is associated with increased risk of RA and BD and is related to a decreased OP risk. Further, each SD increase in Cu can effectively reduce the risk of AD and BD disease.

## 2. Materials and Methods 

### 2.1. Exposure Measures

The summary data for all exposures was downloaded from NHGRI-EBI GWAS (https://www.ebi.ac.uk/gwas), including minerals Ca, Mg, Fe, Cu and Zn.

Ca-related genetic variation was derived from 17 population-based GWAS (*n* = 39,400) results, and an additional 21,679 European ancestry individuals were used to identify relevant genetic loci [16] (Table 1). The genetic variation of Mg is the result of the European ancestry GWAS (*n* = 15,366) from the International CHARGE Alliance, while these SNPs were assessed using an additional 8463 European ancestry individuals [17]. The Fe-related genetic variation was the GWAS result for 11 European populations and was replicated in another 8 populations (total *n* = 48,972) [18]. The genetic variation with Cu and Zn concentrations in erythrocytes were derived from the Queensland Institute of Medicine’s twins and their families (*n* = 2603), which were estimated by using a model adjustment and logarithmic transformation of the genetic association between Cu and Zn, according to a correction that was made within the family [19].

In this study, we chose SNPs with a strong association (*p* < 5 × 10^−8^) and independent inheritance (*r*^2^ < 0.01) without any linkage disequilibrium (LD) from the above mentioned GWAS summary data. European samples from the 1000 Genome Project were used to estimate the LD between the SNPs. When there existed an LD effect among SNPs, we chose the genetic variation with the lowest P value. To assess whether the IV was associated with confounding or risk factors for disease, we used the PhenoScanner (http://www.phenoscanner.medschl.cam.ac.uk/phenoscanner), which provides the SNP phenotype. Information on the minerals is shown in Table 1.

### 2.2. Outcome Measures

Our outcome factors consisted of nine diseases that have been published in relevant GWAS summary data. The first outcome factor was OP, diagnosed largely through measurement of bone mineral density (BMD), characterized by an increased propensity to fracture. The summary-level data of GWAS were obtained from the Genetic Factors for OP (GEFOS, http://www.gefos.org). These data covered 508,253 osteoporotic fractures patients of European ancestry and 53,236 European ancestry individuals in the general population, and three common sites of osteoporotic fractures (femoral neck, lumbar spine, and forearm) were measured, resulting in approximately 10 million summary data points on OP-related SNPs [20]. Our study selected femoral neck data that can corresponded more to mineral-related SNPs as a representative of MR for OP.

The second is gout and the genetic data derived from the Global Urate Genetics Consortium (GUGC). These data are the meta-analysis results of 2115 cases and 67,259 normal individuals from 14 European studies [21]. 

RA is the third outcome factor, and the meta-analysis results of the GWAS for 10 million RA-related SNPs were evaluated in a total of >100,000 subjects of European and Asian ancestry (29,880 RA cases and 73,758 controls) [22]. 

T2D is considered to be the fourth factor. The summary data, which combined three GWAS data sets: DIAbetes Genetics Replication and Meta-analysis (DIAGRAM), Genetic Epidemiology Research on Aging (GERA) and the full cohort release of the UK Biobank (UKB), includes meta-analysis of 62,892 cases of European ancestry and 596,424 normal subjects of European ancestry [23].

The five neurological diseases that were studied included AD, BD, SCZ, PD, and MDD. For AD, the genetic data is from the International Genomics of Alzheimer’s Project (IGAP), which make up of 17,008 patients and 37,154 controls of European ancestry from four GWAS, including the AD Genetic Association (ADGC), the Heart and Aging Research Queue (CHARGE) of the Genome Epidemiology Alliance, the European Alzheimer’s Disease Initiative (EADI), and the genetic and environmental risks of the Alzheimer’s Disease Alliance (GERAD) [24]. For BD, the genetic data is from the newest collection from Psychiatric Genomics Consortium Bipolar Disorder Working Group (PGC-BD). The GWAS analysis was conducted on 20,129 patients and 21,524 controls [25]. For SCZ, the genetic data included a GWAS of 33,426 patients and 32,541 controls of European ancestry [25]. For PD, the genetic data is a meta-analysis of individual level genotypic data from 5 recent PD GWAS (4238 PD and 4239 controls) [26]. For MDD, the dataset is based on a GWAS for 5303 Chinese women with MDD and 5337 controls [27]. All information on the nine diseases is shown in Table 2.

### 2.3. Statistical Analysis for Mendelian Randomization

**Figure 1:**
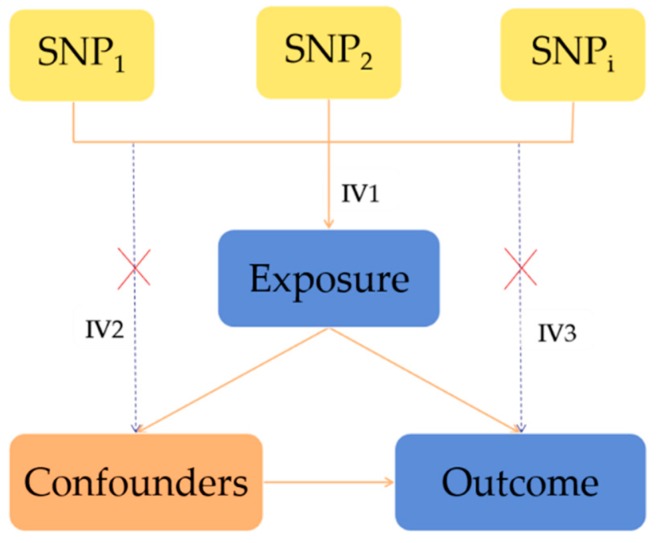
The principle of the MR analysis and the assumptions that need to be met to obtain an unbiased estimate of the causal effect. Instrumental variable (IV) assumption 1: SNPs must be associated with the exposure; IV assumption 2: SNPs must be unrelated to any confounding factors that are associated with the results; IV assumption 3: SNPs must influence the outcome through exposure and not by alternative ways. SNP_1_, SNP_2_, and SNP_i_ = single nucleotide polymorphisms.

The MR method is based on the following InSIDE hypothesis: Genetic variants are associated with the exposure factor; genetic variants must be not related to any confounding factors that are associated with the outcome; genetic variants must influence the outcome through exposure factors rather than through alternative ways (Figure 1). 

There are three methods for MR statistical analysis: inverse variance weighting (IVW), weighted median (WM) and MR-Egger regression. The IVW method yields a consistent causal estimate by combining the Wald ratios of the causal effects of each SNP, but this may also introduce ineffective IVs [28,29]. The WM estimate provides a valid estimate if at least 50% of the weight is from effective IVs [30]. As a sensitivity analysis, we used the MR-Egger method, which can explore and adjust for pleiotropy [31]. However, MR-Egger may be inaccurate, especially when the correlation coefficient between SNPs and the exposure is similar or the number of genetic instruments is small [32]. The WM estimate has the advantage of maintaining a higher estimation accuracy than the MR-Egger method. All statistical tests were two-sided and were considered to show statistical significance at a *P* value below 0.05. The significant causality shown by MR analysis was compared with traditional epidemiology, and the conclusions consistent with traditional investigations provide evidence for MR analysis. Otherwise, they were considered suggestive of evidence for a potential association. The estimate effect value is expressed as the odds ratio (95% confidence interval) and can be recorded as per 1-SD increment in each blood minerals measure with odds ratio (OR) of chronic diseases.

Exposure is considered a changeable risk factor, and we included five minerals as exposure factors, where genetic variants were used as proxies for the exposure. MR analysis predicted the causal relationship between five minerals and nine diseases as genetic evidence. First, we evaluated the independent effects of SNPs that were strongly associated with minerals. Second, the link to SNPs and the potential confounding factors were examined. Third, the causality between blood minerals and diseases was genetically predicted. The analyses were conducted using the “TwoSampleMR” package for R language (version 3.2.3 R Core Team (2017). R: A language and environment for statistical computing. R Foundation for Statistical Computing, Vienna, Austria. URL https://www.R-project.org/).

## 3. Results

### 3.1. Causality between Minerals and Osteoporosis

Six independent SNPs (*p* < 5 × 10^−8^, *r*^2^ < 0.01) were associated with Ca, four independent SNPs were associated with Mg, nine SNPs were associated with Fe, two SNPs were associated with Cu, and two SNPs were associated with Zn by independent and LD analyses (Supplementary Table S2).

**Figure 2:**
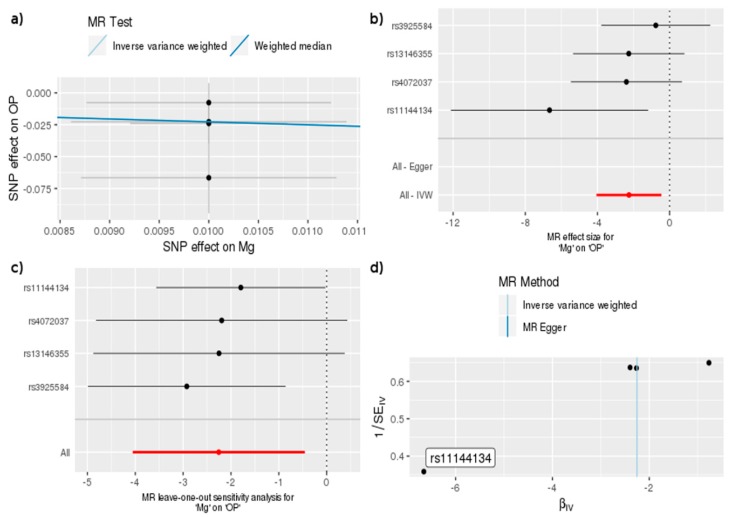
MR study of Mg and OP: (**a**) A graph that correlates the effect size of the SNP-Mg association (x-axis, SD units) and the SNP-OP association (y-axis, log OR) to the standard error bars. The slope of the line corresponds to a causal estimate using a different method. (**b**) The forest map, where each black dot represents a single SNP as IV, shows the logarithm of the odds ratio (OR) per standard deviation (SD) under the influence of mineral magnesium; the red dot shows the use of the IVW results for all SNPs; the horizontal line indicates the 95% confidence interval. (**c**) The leave-one-out method sensitivity analysis. Each black dot represents an IVW method for estimating the causal effect of the Mg element on OP and does not exclude a case where a particular SNP causes a significant change in the overall result. (**d**) The funnel plot shows the estimation using the inverse of the standard error of the causal estimate using each individual SNP as a tool. The vertical line shows the results of the IVW method using all SNPs.

Table 3 shows that the OR of bone density associated with OP per SD (0.16 mmol/L) increase in genetically predicted Mg was a 0.10 g/cm^2^ increase, which means that higher Mg was beneficial in reducing the risk of OP according to the IVW (OR = 0.10, 95% CI = 0.02, 0.63, *p* = 0.014) and WM methods (OR = 0.10, 95% CI = 0.01, 0.82, *p* = 0.032). In addition, by the “single-SNP” and “leave-one-out” methods, we found that one independent SNP (rs11144134) was associated with a significant effect between Mg and OP. This SNP, in the *TRPM6* gene, has also been associated with lower serum Mg and with higher BMD [17], which increases the evidence to support a negative relationship between Mg and OP (Figure 2). 

### 3.2. Causality between Minerals and Gout

We chose seven independent SNPs associated with Ca, five independent SNPs associated with Mg, 11 SNPs associated with Fe, and two SNPs associated with Cu and Zn based on independent and LD analyses (Supplementary Table S3).

Table 4 shows that the OR of gout per SD increase in mineral Mg (0.16 mmol/L) was 0.26 (WM, OR = 0.26, 95% CI = 0.09, 0.76, *p* = 0.013) and per unit increase in mineral Fe was 0.71(MR-Egger, OR = 0.71, 95% CI = 0.53, 0.95, *p* = 0.047), while Ca, Cu, and Zn had no association with the risk of gout. There was no pleiotropy between Mg (MR-Egger regression test, intercept = 0.038, *p* = 0.461), Fe (MR-Egger regression test, intercept = 0.027, *p* = 0.126) and gout. Moreover, the results of the “single-SNP” and “leave-one-out” analyses showed that rs7965584 in the *RP11-654D12.2* gene and rs1800562 in the *HFE* gene corresponded to Mg and Fe, respectively, and had a significant impact on gout (Supplementary Figures S1 and S2).

### 3.3. Causality between Minerals and Rheumatoid Arthritis

We obtained six independent SNPs associated with Ca, four independent SNPs associated with Mg, 11 SNPs associated with Fe, two SNPs associated with Cu, and two SNPs associated with Zn based on independent and LD analyses (Supplementary Table S4).

Table 5 shows that each SD increase in genetically predicted Mg (0.16 mmol/L) was associated with an 8.94-fold increased risk of RA (WM, OR = 8.94, 95% CI = 1.06, 75.70, *p* = 0.044), which means that higher Mg increased the risk of RA. Due to the limitation of multicollinearity, we could not use the MR-egger method to detect the pleiotropic effects. In addition, based on the “single-SNP” and “leave-one-out” methods, we found that rs4072037 in the *MUC1* gene lead to significant effects between Mg and RA (Supplementary Figure S3).

### 3.4. Causality between Minerals and Type 2 Diabetes

Seven independent SNPs were associated with Ca, three independent SNPs were associated with Mg, 10 SNPs were associated with Fe, and two SNPs were associated with Zn (Supplementary Table S5).

Table 6 shows that each SD increase in genetically predicted Ca (0.55 mg/dL) was correlated with an OR for T2D of 1.36 (WM, OR = 1.36, 95%CI = 1.03, 1.79, *p* = 0.03), and each per-unit increase in mineral Fe was correlated with an OR for T2D of 0.89 (IVW, OR = 0.89, 95% CI = 0.81, 0.98, *p* = 0.01; WM, OR = 0.91, 95% CI = 0.86, 0.96, *p* = 5.32× 10^-4^). There was no pleiotropy between Ca (MR-Egger regression test, intercept = −0.04, *p* = 0.09), Fe (MR-Egger regression test, intercept = −0.008, *p* = 0.31) and T2D. Moreover, by the “single-SNP” and “leave-one-out” methods, we found that rs1801725 resulted in a significant effect between Ca and T2D (Supplementary Figure S4), and six independent SNPs (rs1800562, rs4921915, rs174577, rs6486121, rs411988, rs651007) resulted in a significant effect between Fe and T2D (Supplementary Figure S5).

### 3.5. Causality between Minerals and Neurological Diseases

We studied five neurological diseases as outcome factors, including AD, BD, SCZ, PD, and MDD. The SNPs information corresponding to the minerals and the five diseases is shown in Supplementary Tables S6–S10. 

Table 7 shows that each SD increase in genetically predicted Cu was associated with a 0.87-fold increased risk of AD (OR = 0.87, 95%CI = 0.75, 1.00, *p* = 0.05); the OR of BD per SD increase in mineral Mg (0.16 mmol/L) was 8.78 (OR = 8.78, 95%CI = 1.16, 66.26, *p* = 0.04) and per unit increase in mineral Cu was 0.87 (OR = 0.87, 95%CI = 0.79, 0.97, *p* = 0.01).There was no pleiotropy between Fe (MR-Egger regression test, intercept = 0.0005, *p* = 0.97) and BD. In addition, the results of the “single-SNP” and “leave-one-out” methods showed that rs1175550 in the SMIM1 gene significantly affected the correlation of Cu with AD (Supplementary Figure S6); rs4072037 in the *MUC1* gene and rs2769264 in the *SELENBP1* gene, respectively, showed significant effects on the correlation of Mg and Cu with BD (Supplementary Figures S7 and S8). For SCZ, PD and MDD, we found that the five minerals had no causality among them (Table 7).

### 3.6. Causality between Minerals and Nine Diseases

**Figure 3:**
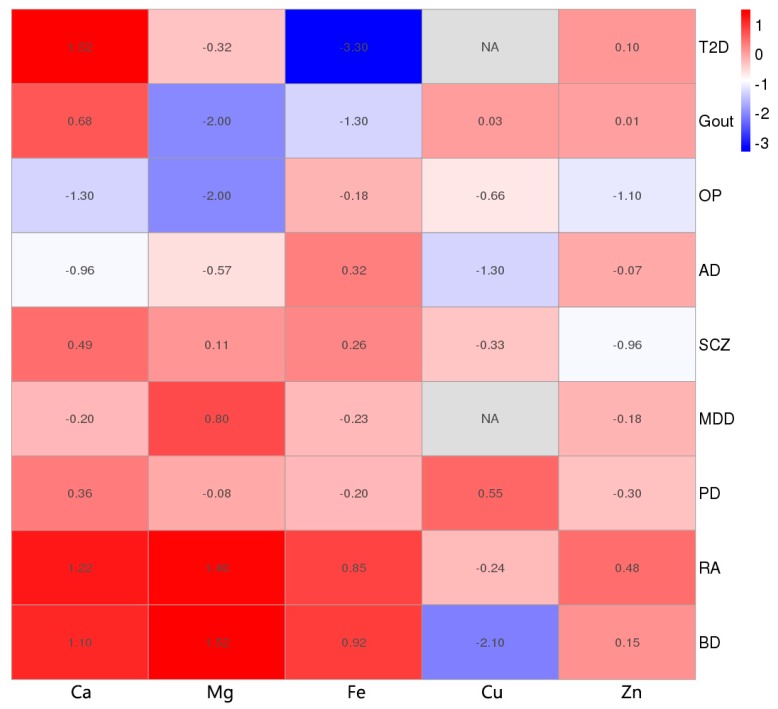
MR estimates of the causality between minerals and diseases in the summary heat map. Red represents a positive correlation, blue represents a negative correlation, and the shade of color represents the significance of the degree of causality. We took the logarithm of the predicted causal effect *p* value, and determined the directionality of the causal relationship according to the positive and negative of the BETA value, with −log (0.05) = 1.30 as the threshold. If the absolute value of each box value is greater than the threshold, it is considered to have a significant causal relationship. The abscissa indicates the five mineral elements, Ca, Mg, Fe, Cu and Zn, and the ordinate indicates the nine diseases. NA indicates a missing value.

Figure 3 shows the causality between five blood minerals and the nine diseases. We found that Ca was positively correlated with T2D only (value = 1.52); Mg was positively correlated with RA (value = 1.4) and BD (value = 1.52) and negatively correlated with gout (value = −2.00) and OP (value = −2.00); Fe was negatively correlated with T2D (value = −3.30); Cu was negatively correlated with BD (value = −2.01); and Zn was not significantly associated with the five diseases.

**Figure 4:**
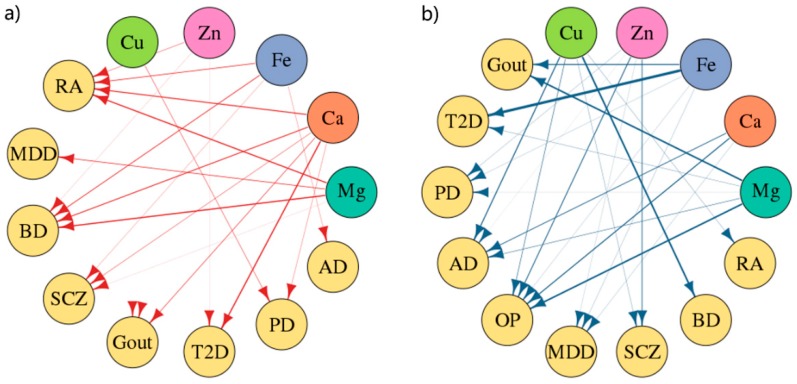
MR estimates of the positive-negative trend causality network diagram of minerals and diseases: (**a**) Positive correlation trend graph. (**b**) Negative correlation trend graph. The line thickness represents the logarithm of the *p* value of the causality: a thicker line represents a more significant *p* value. OP is present only in negative correlation networks because minerals have a negative effect on OP only.

In comparing the results with the results of epidemiological surveys, there were some conclusions that were consistent in two ways, such as that Mg was positively correlated with RA (value = 1.40) [7] and BD (value = 1.52) [33], and negatively correlated with OP (value = −2.00) [34]; Cu was negatively correlated with AD (value = −1.30) [35]. Second, for the epidemiological inconsistency conclusion, such as that Cu has no causality [36] or negative correlation [37] with BD, our research suggested that there was a significantly negative correlation between them (value = −2.10). Third, there are investigations that are inconsistent with our results, such as the result of Ca and T2D (value = 1.52, positive correlation), which are unlike the results of traditional surveys (negative [3] or irrelevant [38]); the causality between Fe and T2D (value = −3.30, negative correlation) is different from the results of traditional surveys (positive [39] or irrelevant [40]). These disparate results may be due to unpredictable confounding factors in traditional research. In addition, for gout diseases without epidemiological findings, our analysis showed a significant negative correlation between Mg and Fe with the risk of gout (value _(Mg)_ = −2.00, value _(Fe)_ = −1.30). Finally, we found some corresponding trend effects. For example, in the traditional epidemiological survey, all five minerals were negatively correlated with the risk of OP, which is consistent with our trend of results. The more obvious trend effect on the positive and negative directions is shown in Figure 4.

## 4. Discussion

Previous epidemiological investigations provided constructive guidance for this study. Our MR results align with those from conventional observational studies wherein each SD increase in genetically predicted Mg (0.16 mmol/L) is associated with an 8.94-fold increased risk of RA [7] and with an 8.74-fold increased risk of BD [33] but with a 0.10 g/cm^2^ increase in bone density related to OP [34]. Each unit increase in Cu was associated with a 0.87-fold increased risk of AD [35] and a 0.87-fold increased risk of BD [37]. 

Physiologically, Mg is the second most abundant intracellular cation and is a co-factor in several important reactions, and serum magnesium concentrations is reportedly associated with several common and chronic diseases [7,17,33,34]. We found strong genetic evidence that rs11144134 in the *TRPM6* gene in our study was associated with lower Mg levels and with higher BMD [17], which increases the evidence to support a negative relationship between Mg and OP. Cu has antioxidant properties, being involved in metabolic processes and redox reactions in the central nervous system [13], and copper affects the evolution of cognitive impairment associated with AD and BD [35,37].

In addition, despite differences from previous epidemiological findings, suggestive evidence indicated that for T2D [3,38,39,40], a per-SD increase in Ca (0.55 mg/dL) and a per-unit increase in Fe were correlated with a 1.36-fold increased risk and a 0.89-fold increased risk, respectively. This difference between the observed evidence and the MR can be explained by confounding factors that were not fully controlled for in observational studies.

Moreover, although there has been no epidemiological study of the relationship between these five minerals and gout, suggestive evidence shows that each SD increase in genetically predicted Mg came with a 0.26-fold increased risk of gout and Fe a 0.71-fold increased risk of gout, which is of great significance for further research on gout disease. Finally, the conclusion of no causality in this study, i.e., that no minerals (Ca, Mg, Fe, Cu, and Zn) were associated with MDD, SCZ, or PD, suggests that previous observations may be the result of these diseases rather than of their prelude.

To take into account multiple testing for the five minerals, we applied a Bonferroni-corrected significance level computed as 0.05 divided by 5 (that is, 0.01). A per-unit increase in Cu was associated with a 0.87-fold change in BD risk, which is strictly significant (*p* = 0.01). However, if there are many covariates, then a hypothesis-testing approach that accounts for the multiple comparisons may lead to a lack of power to detect any specific association. Additionally, as several covariates may be correlated, a simple Bonferroni correction may be an overcorrection [41].

An important advantage of this study is that residual confounding or reverse causality was mitigated through the use of genetic variants as proxies for the mineral level [42]. MR assumes that exposure-related SNPs are independent of the confounder, and that genetic variation affects outcomes only through exposed factors [43]. However, MR studies are susceptible to pleiotropic (i.e., a gene determines or affects the formation of multiple traits) effects [44]. Although the use of a more genetic variation in MR studies increases statistical power, the introduction of an ineffective IV may increase the pleiotropic bias [45]. To eliminate pleiotropic effects, we used MR-Egger regression to test the specificity of the imbalance [32]. The results of this study were consistent in the sensitivity analysis; based on the nonsignificant intercept *p* values generated by MR-Egger, there is no evidence that pleiotropy affects the results.

This study also has certain limitations. First, the three powerful MR assumptions are not empirical. That is, although SNPs that are used as instrumental variables are effective in GWAS, they may increase the likelihood of false positives due to sample size limitations. The presence of weaker IVs can skew the results [46]. Second, the relatively small number of SNPs as IVs can explain only a limited causal relationship [47]. By combining multiple genetic variations, statistical power can be promoted effectively, and more accurate estimates can be obtained [48]. Third, our research population presents complexity, including individuals from Europe, Asia, and other regions. The effects of minerals on humans may depend on race and environment, although we have no reason to believe that they function by population-specific mechanisms. Nevertheless, this inability to reduce these potential impacts in our study results from the population complexity and data uniqueness of the publicly available GWAS related to the five minerals as exposure factors. Further, the use of publicly available data means subgroup analysis by age, sex, and baseline concentrations of five minerals was also not possible. Most importantly, minerals can serve as potential biomarkers for disease, but still no large genetic-level causal analyses have been performed. Therefore, this is an unprecedentedly large-scale MR analysis of the potential role of minerals in the development of various diseases.

## 5. Conclusions

In summary, this study supports the long-standing hypothesis that each 0.16 mmol/L increase in genetically predicted Mg is associated with an 8.94-fold increased risk of RA and an 8.74-fold increased risk of BD, but a 0.10 g/cm^2^ increase in bone density stemming from OP; and each unit increase in genetically predicted Cu is associated with a 0.87-fold increase in the risk of AD and BD. This study will also be helpful to address some controversial debates on the relationships between minerals and chronic diseases. In addition, a well-designed epidemiological combination and MR studies using more IVs can help to further confirm or rule out causality.

## Figures and Tables

**Table 1:** Dataset of the Mineral Information.

Exposure	Number of SNPs	Number of Independent SNPs	Sample Size	Race
Ca	8	8	39,400	Mix + Europe
Mg	5	5	15,366	Europe
Fe	14	11	48,972	Europe
Cu	2	2	2603	Australia
Zn	3	2	2603	Australia

**Table 2:** Information on nine chronic diseases.

	Sample Size			
Outcome	Total	Cases	Controls	Race
OP	561,489	508,253	53,236	Europe
GOUT	69,374	2115	67,259	Europe
RA	103,638	29,880	73,758	Europe
T2D	659,316	62,892	596,424	Europe
AD	54,162	17,008	37,154	Europe
BD	41,653	20,129	21,524	Mix
SCZ	65,967	33,426	32,541	Europe
PD	14,326	7976	6350	Mix
MDD	10,640	5303	5337	Asia

**Table 3:** MR estimates of the causality between five minerals and OP.

							MR-Egger	
Mineral	Number of SNPs	MR Method	OR	95% CI	*p* Value	Cochran’s Q Statistic (*p* Value)	Intercept	Intercept *p* Value
Ca	6	IVW	0.39	0.15, 1.07	0.068	8.02 (0.16)		
		MR-Egger	27.00	0.13, 5581.5	0.292	4.94 (0.29)	−0.080	0.190
		WM	0.37	0.14, 1.02	0.054			
Mg	4	IVW	0.10	0.02, 0.63	0.014	3.43 (0.33)		
		WM	0.10	0.01, 0.82	0.032			
Fe	9	IVW	0.98	0.81, 1.19	0.815	14.78 (0.06)		
		MR-Egger	1.71	0.68, 1.69	0.781	14.39 (0.04)	−0.008	0.680
		WM	0.96	0.80, 1.15	0.661			
Cu	2	IVW	0.93	0.83, 1.04	0.221	1.25 (0.26)		
Zn	2	IVW	0.91	0.82, 1.01	0.077	0.03 (0.86)		

IVW, inverse variance weighting; WM, weighted median; OR, odds ratio.

**Table 4:** MR estimates of the causality between five minerals and gout.

							MR-Egger	
Mineral	Number of SNPs	MR Method	OR	95% CI	*p* Value	Cochran’s Q Statistic (*p* Value)	Intercept	Intercept *p* Value
Ca	7	IVW	2.84	0.45, 17.92	0.267	20.76 (0.002)		
		MR-Egger	1.24	0.04, 38.29	0.908	19.47 (0.002)	0.029	0.589
		WM	2.12	0.66, 6.83	0.206			
Mg	5	IVW	0.33	0.05, 2.08	0.236	15.29 (0.004)		
		MR-Egger	0.15	0.01, 2.09	0.253	12.36 (0.006)	0.038	0.461
		WM	0.26	0.09, 0.76	0.013			
Fe	11	IVW	0.86	0.71, 1.04	0.117	6.34 (0.786)		
		MR-Egger	0.71	0.53, 0.95	0.047	3.50 (0.941)	0.027	0.126
		WM	0.81	0.63, 1.03	0.091			
Cu	2	IVW	1.01	0.80, 1.28	0.928	0.26 (0.607)		
Zn	2	IVW	1.01	0.69. 1.47	0.971	2.77 (0.096)		

**Table 5:** MR estimates of the causality between five minerals and RA.

							MR-Egger	
Mineral	Number of SNPs	MR Method	OR	95% CI	*p* Value	Cochran’s Q Statistic (*p* Value)	Intercept	Intercept *p* Value
Ca	6	IVW	1.83	0.99, 3.41	0.055	3.40 (0.638)		
		MR-Egger	2.42	0.80, 7.35	0.194	3.05 (0.549)	−0.01	0.59
		WM	1.93	0.94, 3.94	0.073			
Mg	4	IVW	3.07	0.16, 58.62	0.457	8.11 (0.044)		
		WM	8.94	1.06, 75.70	0.044			
Fe	11	IVW	1.01	0.82, 1.25	0.913	42.22 (6.84 × 10^−6^)		
		MR-Egger	1.19	0.90, 1.57	0.262	33.21 (1.23 × 10^−4^)	−0.03	0.15
		WM	1.12	0.97, 1.29	0.138			
Cu	2	IVW	0.94	0.77, 1.16	0.579	2.26 (0.13)		
Zn	2	IVW	1.07	0.94, 1.22	0.328	0.54 (0.46)		

**Table 6:** MR estimates of the causality between five minerals and T2D.

							MR-Egger	
Mineral	Number of SNPs	MR Method	OR	95% CI	*p* Value	Cochran’s Q Statistic (*p* Value)	Intercept	Intercept *p* Value
Ca	7	IVW	0.88	0.34, 2.27	0.80	99.35 (3.43 × 10^−19^)		
		MR-Egger	2.82	0.75, 10.58	0.19	52.98 (3.40 × 10^−10^)	−0.040	0.090
		WM	1.36	1.03, 1.79	0.03			
Mg	3	IVW	1.55	0.26, 9.24	0.63	7.99 (0.02)		
		WM	0.64	0.18, 2.22	0.48			
Fe	10	IVW	0.89	0.81, 0.98	0.01	41.06 (4.87 × 10^−6^)		
		MR-Egger	0.93	0.82, 1.07	0.36	35.87 (1.85 × 10^−5^)	−0.008	0.310
		WM	0.91	0.86, 0.96	5.32 × 10^−4^			
Zn	2	IVW	1.01	0.92, 1.12	0.79	3.77 (0.05)		

**Table 7:** MR estimates of the causality between five minerals and five neurologic diseases.

**Outcome: AD**								
							**MR-Egger**	
**Mineral**	**Number of SNPs**	**MR Method**	**OR**	**95% CI**	***p* Value**	**Cochran’s** **Q Statistic** **(*p* Value)**	**Intercept**	**Intercept *p* Value**
Ca	6	IVW	0.74	0.45, 1.22	0.23	4.08 (0.54)		
		MR-Egger	0.46	0.19, 1.14	0.17	2.62 (0.62)	0.020	0.29
		WM	0.64	0.37, 1.10	0.11			
Mg	4	IVW	0.43	0.08, 2.44	0.34	1.16 (0.76)		
		WM	0.30	0.04, 2.53	0.27			
Fe	11	IVW	1.04	0.94, 1.14	0.48	8.35 (0.59)		
		MR-Egger	1.02	0.88, 1.17	0.82	8.24 (0.51)	0.005	0.75
		WM	1.03	0.92, 1.15	0.62			
Cu	2	IVW	0.87	0.75, 1.00	0.05	1.72 (0.20)		
Zn	2	IVW	0.99	0.85, 1.14	0.85	1.96 (0.16)		
**Outcome: BD**								
							**MR-Egger**	
**Mineral**	**Number of SNPs**	**MR Method**	**OR**	**95% CI**	***p* Value**	**Cochran’s** **Q Statistic** **(*p* Value)**	**Intercept**	**Intercept *p* Value**
Ca	7	IVW	1.85	0.74, 4.65	0.19	23.99 (5.25 × 10^−4^)		
		MR-Egger	1.27	0.22, 7.29	0.80	22.79 (3.70 × 10^−4^)	0.013	0.63
		WM	1.63	0.94, 2.82	0.08			
Mg	4	IVW	8.78	1.16, 66.26	0.04	4.66 (0.198)		
		WM	8.02	0.91, 70.43	0.06			
Fe	11	IVW	1.07	0.89, 1.29	0.45	41.0 (1.13 × 10^−5^)		
		MR-Egger	1.07	0.8, 1.43	0.66	40.99 (5.01 × 10^−6^)	0.0005	0.97
		WM	1.10	0.98, 1.23	0.12			
Cu	2	IVW	0.87	0.79, 0.97	0.01	0.15 (0.70)		
Zn	2	IVW	1.02	0.91, 1.14	0.70	1.24 (0.27)		
**Outcome: SCZ**								
							**MR-Egger**	
**Mineral**	**Number of SNPs**	**MR Method**	**OR**	**95% CI**	***p* Value**	**Cochran’s** **Q Statistic** **(*p* Value)**	**Intercept**	**Intercept *p* Value**
Ca	7	IVW	0.81	0.53, 1.23	0.32	7.48 (0.28)		
		MR-Egger	1.05	0.49, 2.25	0.91	6.63 (0.25)	−0.009	0.46
		WM	0.93	0.6, 1.45	0.75			
Mg	4	IVW	0.87	0.24, 3.19	0.83	2.31 (0.51)		
		WM	0.79	0.15, 4.07	0.77			
Fe	10	IVW	1.04	0.92, 1.18	0.55	12.6 (0.18)		
		MR-Egger	0.91	0.69, 1.22	0.55	11.27 (0.19)	0.01	0.36
		WM	1.01	0.88, 1.16	0.85			
Cu	2	IVW	0.96	0.85, 1.08	0.47	2.22 (0.14)		
Zn	2	IVW	0.94	0.86, 1.02	0.11	1.02 (0.31)		
**Outcome: PD**								
							**MR-Egger**	
**Mineral**	**Number of SNPs**	**MR Method**	**OR**	**95% CI**	***p* Value**	**Cochran’s** **Q Statistic** **(*p* Value)**	**Intercept**	**Intercept *p* Value**
Ca	7	IVW	1.57	0.49, 5.02	0.44	8.05 (0.23)		
		MR-Egger	1.34	0.14, 12.65	0.81	8.01 (0.16)	0.005	0.87
		WM	1.53	0.47, 4.95	0.48			
Mg	5	IVW	0.92	0.35, 2.42	0.86	1.32 (0.86)		
		MR-Egger	0.96	0.26, 3.59	0.95	1.31 (0.73)	−0.002	0.93
		WM	0.90	0.32, 2.52	0.84			
Fe	11	IVW	0.95	0.78, 1.16	0.63	10.17 (0.43)		
		MR-Egger	0.99	0.72, 1.35	0.94	10.06 (0.35)	−0.005	0.76
		WM	1.06	0.82, 1.36	0.65			
Cu	2	IVW	1.13	0.91,1.41	0.28	0.39 (0.53)		
Zn	2	IVW	0.92	0.71,1.18	0.50	1.36 (0.24)		
**Outcome: MDD**								
							**MR-Egger**	
**Mineral**	**Number of SNPs**	**MR Method**	**OR**	**95% CI**	***p* Value**	**Cochran’s** **Q Statistic** **(*p* Value)**	**Intercept**	**Intercept *p* Value**
Ca	6	IVW	0.92	0.67, 1.28	0.63	3.54 (0.62)		
		MR-Egger	1.16	0.56, 2.38	0.71	3.06 (0.55)	−0.005	0.53
		WM	1.01	0.68, 1.51	0.95			
Mg	3	IVW	1.19	0.22, 6.61	0.84	7.06 (0.03)		
		WM	2.25	0.72, 7.06	0.17			
Fe	9	IVW	0.98	0.91, 1.05	0.60	1.57 (0.99)		
		MR-Egger	0.98	0.83, 1.02	0.85	1.57 (0.98)	−0.0002	0.97
		WM	0.98	0.9, 1.07	0.72			
Zn	2	IVW	0.99	0.95, 1.03	0.66	0.004 (0.95)		

## Supplementary Materials

The following are available online at https://www.mdpi.com/2072-6643/11/2/378/s1; Figure S1: MR study of Mg and Gout; Figure S2: MR study of Fe and Gout; Figure S3: MR study of Mg and RA; Figure S4: MR study of Ca and T2D; Figure S5: MR study of Fe and T2D; Figure S6: MR study of Cu and AD; Figure S7: MR study of Mg and BD; Figure S8: MR study of Cu and BD; Table S1: Summary of the relationship between mineral nutrition and diseases in traditional epidemiological surveys; Table S2: Summary statistics for the genetic variants associated with the exposure factors investigated for an association with osteoporosis in the present Mendelian randomization study; Table S3: Summary statistics for the genetic variants associated with the exposure factors investigated for an association with gout in the present Mendelian randomization study; Table S4: Summary statistics for the genetic variants associated with the exposure factors investigated for an association with RA in the present Mendelian randomization study; Table S5: Summary statistics for the genetic variants associated with the exposure factors investigated for an association with T2D in the present Mendelian randomization study; Table S6: Summary statistics for the genetic variants associated with the exposure factors investigated for an association with AD in the present Mendelian randomization study; Table S7: Summary statistics for the genetic variants associated with the exposure factors investigated for an association with BD in the present Mendelian randomization study; Table S8: Summary statistics for the genetic variants associated with the exposure factors investigated for an association with SCZ in the present Mendelian randomization study; Table S9: Summary statistics for the genetic variants associated with the exposure factors investigated for an association with PD in the present Mendelian randomization study; Table S10: Summary statistics for the genetic variants associated with the exposure factors investigated for an association with MDD in the present Mendelian randomization study; Gene abbreviation.
