# Supplementary material for: Mineral Nutrition and the Risk of Chronic Diseases: A Mendelian Randomization Study
Source: Nutrients. 2019 Feb 12;11(2):378. doi: 10.3390/nu11020378 (PMC6412267; doi:10.3390/nu11020378)
Supplement: Supplementary file 1 [file nutrients-11-00378-s001.pdf]

Supplementary data

Supplementary Table 1. Summary of the relationship between mineral nutrition and diseases in traditional epidemiological surveys.

Reference	Mineral	Disease	The relationship between minerals and diseases
Bristow et al, 2017 [1]	Ca	OP	Negative
Welch et al, 2017 [2]	Mg	OP	Negative
Kunutsor et al, 2017 [3]	Mg	OP	Negative
Finck et al, 2015 [4]	Fe	OP	Negative
Kim et al, 2016 [5]	Zn	OP	Negative
Qu et al, 2018 [6]	Cu	OP	Negative
Liu et al, 2017 [7]	Ca	RA	Positive
dos Santos et al, 2016 [8]	Mg, Zn, and Cu	RA	All positive
Garcia et al, 2007 [9]	Fe	RA	Irrelevant
Cerhan et al, 2003 [10]	Zn and Cu	RA	Negative
Squitti et al,2017 [11]	Cu	T2D	Positive
Fang et al, 2016 [12]	Mg	T2D	Negative
Kim et al, 2018 [13]	Ca	T2D	Irrelevant
Villegas et al, 2009 [14]	Ca and Mg	T2D	Negative
Drake et al, 2017 [15]	Zn	T2D	Negative
Podmore et al, 2016 [16]	Fe	T2D	Irrelevant
Zhao et al, 2012 [17]	Fe	T2D	Positive
Cherbuin et al, 2014 [18]	Mg and Fe	AD	Negative, positive, respectively
Ozawa et al, 2012 [19]	Ca and Mg	AD	All negative
Strozyk et al, 2009 [20]	Cu and Zn	AD	All negative
Rembach et al, 2014 [21]	Zn	AD	Irrelevant
Smorgon et al, 2004 [22]	Cu and Fe	AD	Negative, positive, respectively
Siwek et al, 2015 [23]	Mg	BD	Positive
Siwek et al, 2017 [24]	Cu	BD	Irrelevant
Styczen et al, 2018 [25]	Cu and Zn	BD, MDD	Irrelevant
Gonzalez et al, 2011[26]	Zn	BD	Positive
Nielsen et al, 2016 [27]	Fe	SCZ	Negative
Vidovic et al, 2013 [28]	Cu and Zn	SCZ	Positively, irrelevant, respectively
Miyake et al, 2011 [29]	Ca	PD	Irrelevant
Zhao et al, 2013 [30]	Se, Fe, Zn, and Cu	PD	Positive, positive, negative, negative
Hong et al, 2016 [31]	Fe	PD	Irrelevant
Jacka et al, 2009 [32]	Mg	MDD	Negative
Mills et al, 2017 [33]	Fe	MDD	Irrelevant
Li et al, 2017 [34]	Zn and Fe	MDD	Negative
Whitfield et al, 2015 [35]	Zn	MDD	Negative
Roy et al, 2010 [36]	Zn	MDD	Negative

Abbreviations: Negative, mineral supplementation is associated with reduced disease risk; Positive, mineral supplementation is associated with increased disease risk; Irrelevant, there is no causal relationship between mineral supplementation and disease risk; Ca, calcium; Mg, magnesium; Fe, iron; Cu, copper; Zn, zinc; OP, osteoporosis; RA, rheumatoid arthritis; T2D, type 2 diabetes; AD, Alzheimer's disease; BD, bipolar disorder; SCZ, schizophrenia; PD,

Parkinson's disease; MDD, major depressive disorder.

Supplementary Table 2. Summary statistics for the genetic variants associated with the exposure factors investigated for an association with osteoporosis in the present Mendelian randomization study.

Exp	SNP	Gene	Effect_ allele	Other_ allele	Beta_ Exp	Se_ Exp	Pval_ Exp	Beta_ Out	Se_ Out	Pval_ Out
Ca	rs10491003	*LINC00709*	T	C	0.027	0.005	2.51E-09	0.037	0.027	0.085
Ca	rs1550532	*DGKD*	C	G	0.018	0.003	2.97E-11	-0.003	0.017	0.418
Ca	rs1570669	*CYP24A1*	G	A	0.018	0.003	4.53E-12	-0.017	0.016	0.142
Ca	rs17711722	*GTF2IP5*	T	C	0.015	0.003	3.98E-09	-0.010	0.019	0.297
Ca	rs7336933	*VWA8-AS1*	G	A	0.022	0.004	4.49E-10	-0.036	0.022	0.047
Ca	rs7481584	*CARS*	G	A	0.018	0.003	4.97E-11	-0.018	0.017	0.143
Ca	rs780094	*GCKR*	T	C	0.017	0.003	5.02E-11	-0.039	0.016	0.007
Mg	rs11144134	*TRPM6*	C	T	0.01	0.001	3.92E-15	-0.067	0.028	0.009
Mg	rs13146355	*SHROOM3*	A	G	0.01	0.001	3.02E-13	-0.023	0.016	0.075
Mg	rs3925584	*DCDC1*	T	C	0.01	0.001	2.52E-16	-0.008	0.015	0.309
Mg	rs4072037	*MUC1*	T	C	0.01	0.001	1.00E-36	-0.024	0.016	0.064
Fe	rs174577	*FADS2*	A	C	0.062	0.007	9.96E-18	0.040	0.016	0.007
Fe	rs411988	*TEX14*	G	A	0.044	0.007	1.00E-10	-0.019	0.016	0.123
Fe	rs6486121	*ARNTL*	C	T	0.046	0.007	2.00E-10	-0.007	0.016	0.341
Fe	rs651007	*ABO*	C	T	0.05	0.009	5.00E-09	-0.027	0.018	0.070
Fe	rs7385804	*TFR2*	A	C	0.064	0.007	5.01E-19	0.025	0.016	0.061
Fe	rs744653	*AC013439.4*	C	T	0.089	0.01	4.16E-19	-0.006	0.022	0.394
Fe	rs8177179	*TF*	G	A	0.154	0.01	1.52E-49	-0.005	0.016	0.386
Fe	rs855791	*TMPRSS6*	G	A	0.055	0.007	5.02E-15	-0.024	0.016	0.062
Fe	rs9990333	*TFRC*	C	T	0.051	0.007	1.00E-13	-0.009	0.016	0.273
Cu	rs1175550	*SMIM1*	G	A	0.198	0.032	2.51E-10	0.003	0.018	0.442
Cu	rs2769264	*SELENBP1*	G	T	0.313	0.034	1.49E-20	-0.035	0.020	0.039
Zn	rs1532423	*CA1*	A	G	0.178	0.026	2.98E-12	-0.019	0.016	0.113
Zn	rs2120019	*PPCDC*	T	C	0.287	0.033	9.94E-19	-0.026	0.020	0.096

Abbreviations: Exp, exposure factor; Beta_Exp, the effect size exposure factor; Se_Exp, the standard error in the effect size of exposure factors; Pval_Exp, the P-value for the SNP's association with the exposure; Beta_Out, the effect size outcome; Se_Out, the standard error in the effect size of outcome; Pval_Out, the P-value for the SNP's association with the outcome.

Supplementary Figure 1. MR study of Mg and gout.

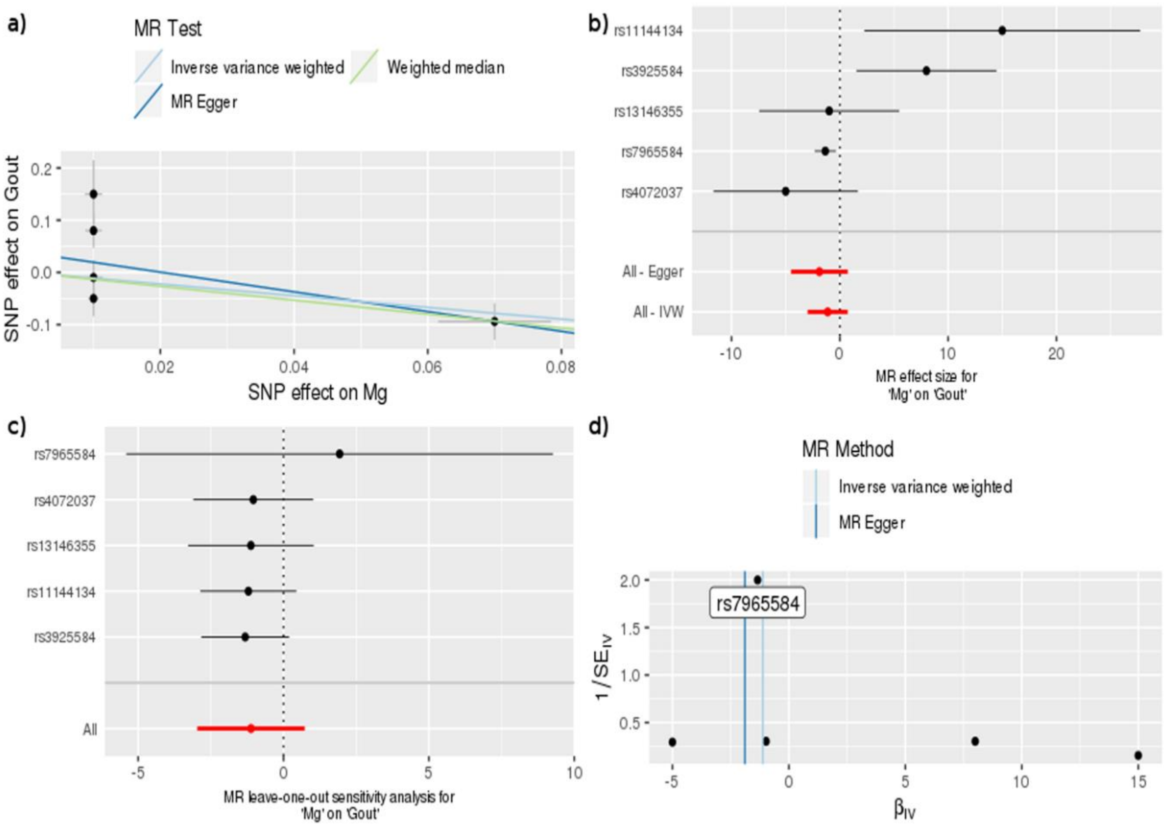

Supplementary Figure 2. MR study of Fe and gout.

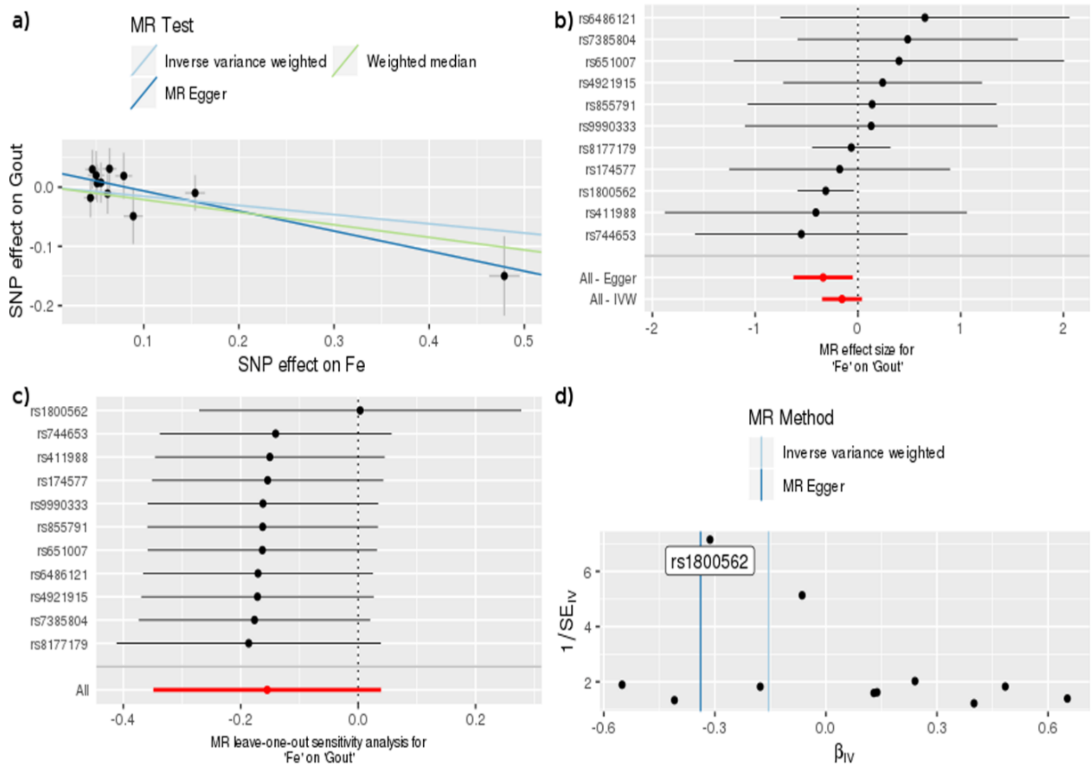

Supplementary Table 3. Summary statistics for the genetic variants associated with the exposure factors investigated for an association with gout in the present Mendelian randomization study.

Exp	SNP	Gene	Effect_ allele	Other_ allele	Beta_ exp	Se_ exp	Pval_ exp	Beta_ out	Se_ out	Pval_ out
Ca	rs10491003	*LINC00709*	T	C	0.027	0.005	2.51E-09	-0.079	0.060	0.094
Ca	rs1550532	*DGKD*	C	G	0.018	0.003	2.97E-11	-0.005	0.034	0.446
Ca	rs1570669	*CYP24A1*	G	A	0.018	0.003	4.53E-12	-0.008	0.034	0.410
Ca	rs17711722	*GTF2IP5*	T	C	0.015	0.003	3.98E-09	-0.026	0.034	0.222
Ca	rs1801725	*CASR*	T	G	0.071	0.004	4.33E-86	0.054	0.045	0.115
Ca	rs7336933	*VWA8-AS1*	G	A	0.022	0.004	4.49E-10	0.026	0.046	0.286
Ca	rs7481584	*CARS*	G	A	0.018	0.003	4.97E-11	0.049	0.037	0.093
Ca	rs780094	*GCKR*	T	C	0.017	0.003	5.02E-11	0.140	0.032	0.000
Mg	rs11144134	*TRPM6*	C	T	0.01	0.001	3.92E-15	0.150	0.065	0.011
Mg	rs13146355	*SHROOM3*	A	G	0.01	0.001	3.02E-13	-0.010	0.033	0.383
Mg	rs3925584	*DCDC1*	T	C	0.01	0.001	2.52E-16	0.080	0.033	0.008
Mg	rs4072037	*MUC1*	T	C	0.01	0.001	1.00E-36	-0.050	0.034	0.071
Mg	rs7965584	*RP11-654D12.2*	A	G	0.07	0.008	5.01E-17	-0.094	0.035	0.004
Fe	rs174577	*FADS2*	A	C	0.062	0.007	9.96E-18	-0.011	0.034	0.373
Fe	rs1800562	*HFE*	G	A	0.479	0.016	5.21E-196	-0.150	0.067	0.013
Fe	rs411988	*TEX14*	G	A	0.044	0.007	1.00E-10	-0.018	0.033	0.293
Fe	rs4921915	*NAT2*	A	G	0.079	0.009	3.52E-19	0.019	0.039	0.313
Fe	rs6486121	*ARNTL*	C	T	0.046	0.007	2.00E-10	0.030	0.033	0.182
Fe	rs651007	*ABO*	C	T	0.05	0.009	5.00E-09	0.020	0.041	0.313
Fe	rs7385804	*TFR2*	A	C	0.064	0.007	5.01E-19	0.031	0.035	0.188
Fe	rs744653	*AC013439.4*	C	T	0.089	0.01	4.16E-19	-0.049	0.047	0.149
Fe	rs8177179	*TF*	G	A	0.154	0.01	1.52E-49	-0.010	0.030	0.371
Fe	rs855791	*TMPRSS6*	G	A	0.055	0.007	5.02E-15	0.008	0.034	0.412
Fe	rs9990333	*TFRC*	C	T	0.051	0.007	1.00E-13	0.007	0.032	0.418
Cu	rs1175550	*SMIM1*	G	A	0.198	0.032	2.51E-10	0.020	0.042	0.317
Cu	rs2769264	*SELENBP1*	G	T	0.313	0.034	1.49E-20	-0.010	0.045	0.416
Zn	rs1532423	*CA1*	A	G	0.178	0.026	2.98E-12	0.044	0.033	0.091
Zn	rs2120019	*PPCDC*	T	C	0.287	0.033	9.94E-19	-0.043	0.043	0.159

Supplementary Figure 3. MR study of Mg and RA.

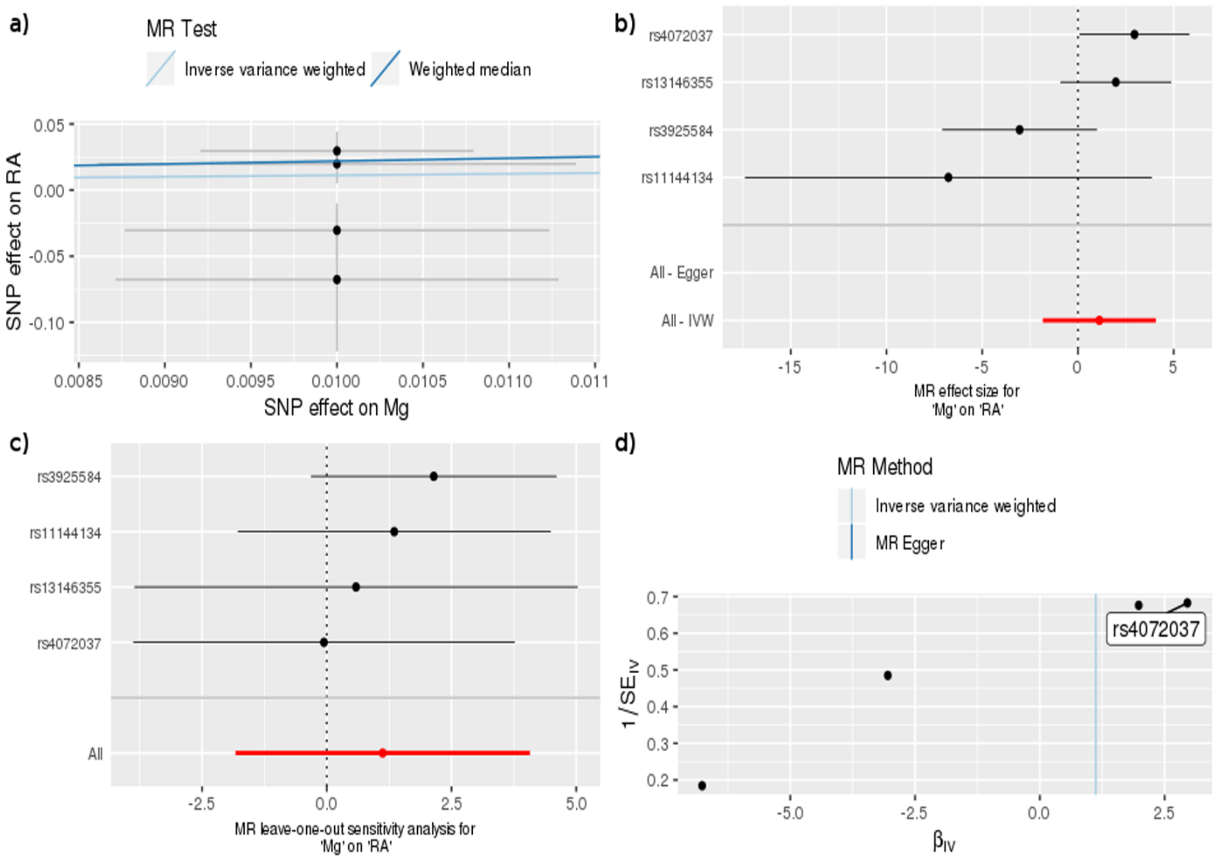

Supplementary Table 4. Summary statistics for the genetic variants associated with the exposure factors investigated for an association with RA in the present Mendelian randomization study.

Exp	SNP	Gene	Effect_ allele	Other_ allele	Beta_ exp	Se_ exp	Pval_ exp	Beta_ out	Se_ out	Pval_ out
Ca	rs10491003	*LINC00709*	T	C	0.027	0.005	2.51E-09	-0.016	0.028	0.284
Ca	rs1550532	*DGKD*	C	G	0.018	0.003	2.97E-11	-0.021	0.017	0.109
Ca	rs1570669	*CYP24A1*	G	A	0.018	0.003	4.53E-12	-0.006	0.017	0.364
Ca	rs1801725	*CASR*	T	G	0.071	0.004	4.33E-86	-0.036	0.023	0.057
Ca	rs7336933	*VWA8-AS1*	G	A	0.022	0.004	4.49E-10	-0.005	0.022	0.414
Ca	rs7481584	*CARS*	G	A	0.018	0.003	4.97E-11	0.027	0.017	0.058
Ca	rs780094	*GCKR*	T	C	0.017	0.003	5.02E-11	-0.003	0.016	0.433
Mg	rs11144134	*TRPM6*	C	T	0.010	0.001	3.92E-15	0.022	0.031	0.237
Mg	rs13146355	*SHROOM3*	A	G	0.010	0.001	3.02E-13	-0.014	0.016	0.187
Mg	rs3925584	*DCDC1*	T	C	0.010	0.001	2.52E-16	-0.012	0.016	0.222
Mg	rs4072037	*MUC1*	T	C	0.010	0.001	1.00E-36	-0.007	0.017	0.338
Fe	rs174577	*FADS2*	A	C	0.062	0.007	9.96E-18	-0.010	0.016	0.279
Fe	rs1800562	*HFE*	G	A	0.479	0.016	5.21E-196	0.017	0.033	0.301
Fe	rs411988	*TEX14*	G	A	0.044	0.007	1.00E-10	0.032	0.015	0.019
Fe	rs4921915	*NAT2*	A	G	0.079	0.009	3.52E-19	0.004	0.019	0.421
Fe	rs6486121	*ARNTL*	C	T	0.046	0.007	2.00E-10	-0.023	0.017	0.081
Fe	rs651007	*ABO*	C	T	0.050	0.009	5.00E-09	0.001	0.019	0.479
Fe	rs7385804	*TFR2*	A	C	0.064	0.007	5.01E-19	0.016	0.017	0.165
Fe	rs744653	*AC013439.4*	C	T	0.089	0.010	4.16E-19	-0.004	0.023	0.427
Fe	rs8177179	*TF*	G	A	0.154	0.010	1.52E-49	0.002	0.016	0.454
Fe	rs855791	*TMPRSS6*	G	A	0.055	0.007	5.02E-15	-0.004	0.016	0.398
Fe	rs9990333	*TFRC*	C	T	0.051	0.007	1.00E-13	0.015	0.017	0.183
Cu	rs1175550	*SMIM1*	G	A	0.198	0.032	2.51E-10	-0.049	0.019	0.005
Cu	rs2769264	*SELENBP1*	G	T	0.313	0.034	1.49E-20	-0.029	0.021	0.083
Zn	rs1532423	*CA1*	A	G	0.178	0.026	2.98E-12	-0.020	0.016	0.102
Zn	rs2120019	*PPCDC*	T	C	0.287	0.033	9.94E-19	0.013	0.020	0.259

Supplementary Figure 4. MR study of Ca and T2D.

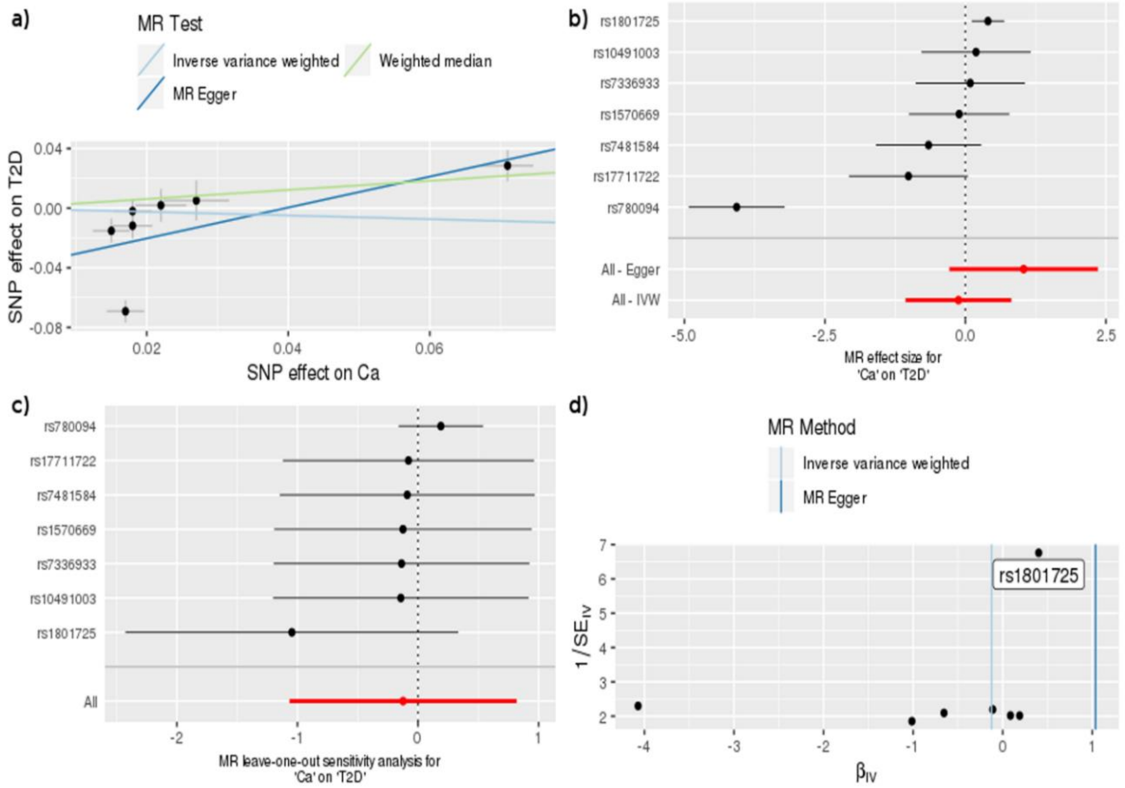

Supplementary Figure 5. MR study of Fe and T2D.

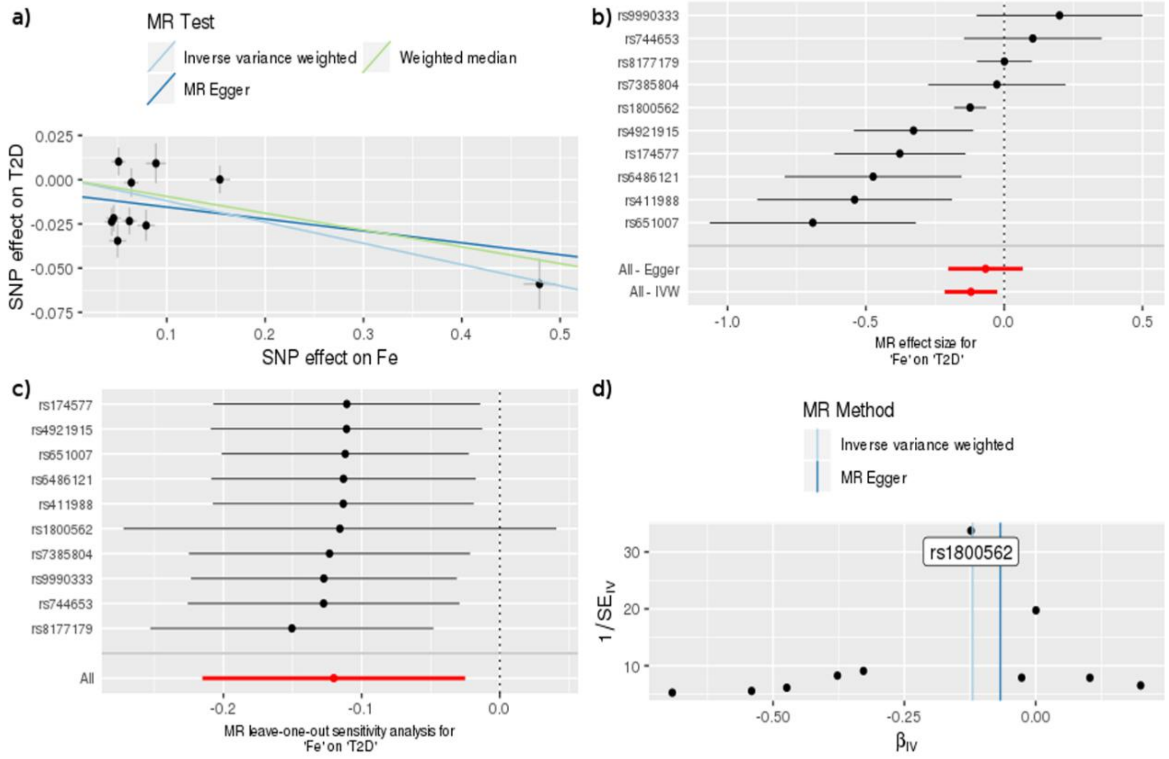

Supplementary Table 5. Summary statistics for the genetic variants associated with the exposure factors investigated for an association with T2D in the present Mendelian randomization study.

Exp	SNP	Gene	Effect_ allele	Other_ allele	Beta_ exp	Se_ exp	Pval_ exp	Beta_ out	Se_ out	Pval_ out
Ca	rs10491003	*LINC00709*	T	C	0.027	0.005	2.51E-09	0.005	0.013	0.352
Ca	rs1550532	*DGKD*	C	G	0.018	0.003	2.97E-11	-0.010	0.008	0.124
Ca	rs1570669	*CYP24A1*	G	A	0.018	0.003	4.53E-12	-0.002	0.008	0.404
Ca	rs17711722	*GTF2IP5*	T	C	0.015	0.003	3.98E-09	-0.015	0.008	0.030
Ca	rs1801725	*CASR*	T	G	0.071	0.004	4.33E-86	0.029	0.011	0.003
Ca	rs7336933	*VWA8-AS1*	G	A	0.022	0.004	4.49E-10	0.002	0.011	0.431
Ca	rs7481584	*CARS*	G	A	0.018	0.003	4.97E-11	-0.012	0.009	0.085
Ca	rs780094	*GCKR*	T	C	0.017	0.003	5.02E-11	-0.069	0.007	4.33E-21
Mg	rs13146355	*SHROOM3*	A	G	0.010	0.001	3.02E-13	-0.005	0.008	0.263
Mg	rs3925584	*DCDC1*	T	C	0.010	0.001	2.52E-16	-0.005	0.008	0.284
Mg	rs4072037	*MUC1*	T	C	0.010	0.001	1.00E-36	0.023	0.008	0.002
Fe	rs174577	*FADS2*	A	C	0.062	0.007	9.96E-18	-0.023	0.008	0.001
Fe	rs1800562	*HFE*	G	A	0.479	0.016	5.21E-196	-0.059	0.014	1.63E-05
Fe	rs411988	*TEX14*	G	A	0.044	0.007	1.00E-10	-0.024	0.008	0.001
Fe	rs4921915	*NAT2*	A	G	0.079	0.009	3.52E-19	-0.026	0.009	0.001
Fe	rs6486121	*ARNTL*	C	T	0.046	0.007	2.00E-10	-0.022	0.008	0.002
Fe	rs651007	*ABO*	C	T	0.050	0.009	5.00E-09	-0.035	0.010	0.000
Fe	rs7385804	*TFR2*	A	C	0.064	0.007	5.01E-19	-0.002	0.008	0.417
Fe	rs744653	*AC013439.4*	C	T	0.089	0.010	4.16E-19	0.009	0.011	0.208
Fe	rs8177179	*TF*	G	A	0.154	0.010	1.52E-49	1.00E-04	0.008	0.495
Fe	rs9990333	*TFRC*	C	T	0.051	0.007	1.00E-13	0.010	0.008	0.095
Cu	rs2769264	*SELENBP1*	G	T	0.313	0.034	1.49E-20	0.024	0.010	0.009
Zn	rs1532423	*CA1*	A	G	0.178	0.026	2.98E-12	-0.010	0.008	0.106
Zn	rs2120019	*PPCDC*	T	C	0.287	0.033	9.94E-19	0.015	0.010	0.058

Supplementary Figure 6. MR study of Cu and AD.

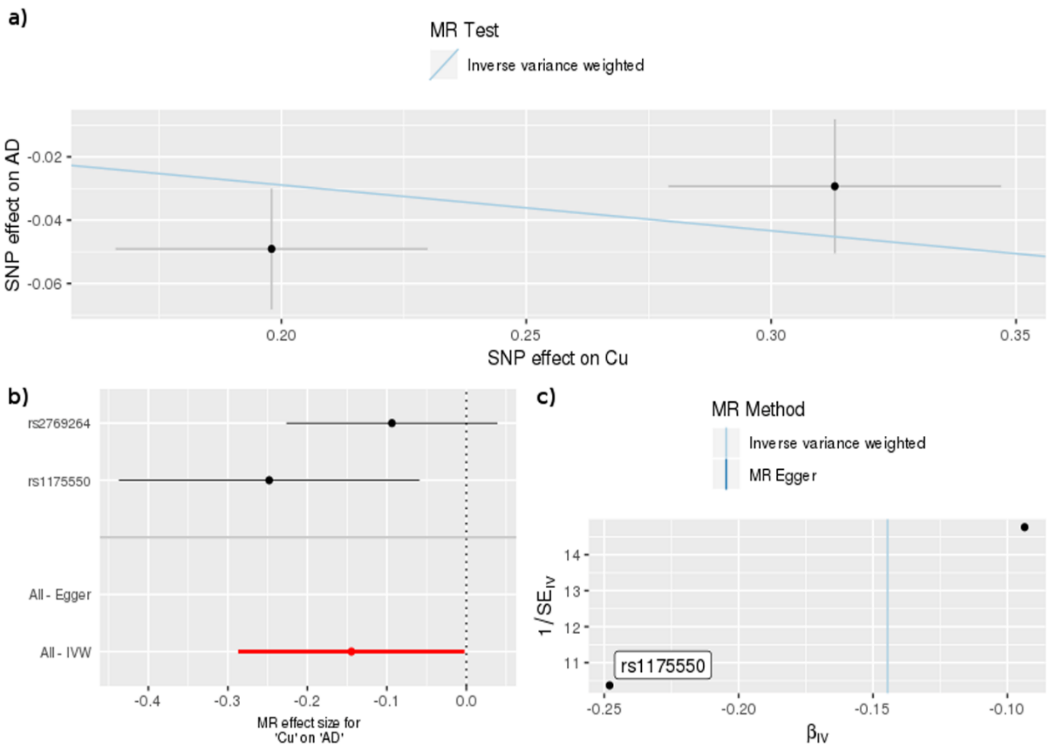

Supplementary Table 6. Summary statistics for the genetic variants associated with the exposure factors investigated for an association with AD in the present Mendelian randomization study.

Exp	SNP	Gene	Effect_ allele	Other_ allele	Beta_ exp	Se_ exp	Pval_ exp	Beta_ out	Se_ out	Pval_ out
Ca	rs10491003	*LINC00709*	T	C	0.03	0.005	2.51E-09	-0.02	0.03	0.28
Ca	rs1550532	*DGKD*	C	G	0.02	0.003	2.97E-11	-0.02	0.02	0.11
Ca	rs1570669	*CYP24A1*	G	A	0.02	0.003	4.53E-12	-0.01	0.02	0.36
Ca	rs1801725	*CASR*	T	G	0.07	0.004	4.33E-86	-0.04	0.02	0.06
Ca	rs7336933	*VWA8-AS1*	G	A	0.02	0.004	4.49E-10	0.00	0.02	0.41
Ca	rs7481584	*CARS*	G	A	0.02	0.003	4.97E-11	0.03	0.02	0.06
Ca	rs780094	*GCKR*	T	C	0.02	0.003	5.02E-11	0.00	0.02	0.43
Mg	rs11144134	*TRPM6*	C	T	0.01	0.001	3.92E-15	0.02	0.03	0.24
Mg	rs13146355	*SHROOM3*	A	G	0.01	0.001	3.02E-13	-0.01	0.02	0.19
Mg	rs3925584	*DCDC1*	T	C	0.01	0.001	2.52E-16	-0.01	0.02	0.22
Mg	rs4072037	*MUC1*	T	C	0.01	0.001	1E-36	-0.01	0.02	0.34
Fe	rs174577	*FADS2*	A	C	0.06	0.007	9.96E-18	-0.01	0.02	0.28
Fe	rs1800562	*HFE*	G	A	0.48	0.016	5.21E-196	0.02	0.03	0.30
Fe	rs411988	*TEX14*	G	A	0.04	0.007	1E-10	0.03	0.02	0.02
Fe	rs4921915	*NAT2*	A	G	0.08	0.009	3.52E-19	0.00	0.02	0.42
Fe	rs6486121	*ARNTL*	C	T	0.05	0.007	2E-10	-0.02	0.02	0.08
Fe	rs651007	*ABO*	C	T	0.05	0.009	5E-9	0.00	0.02	0.48
Fe	rs7385804	*TFR2*	A	C	0.06	0.007	5.01E-19	0.02	0.02	0.16
Fe	rs744653	*AC013439.4*	C	T	0.09	0.01	4.16E-19	0.00	0.02	0.43
Fe	rs8177179	*TF*	G	A	0.15	0.01	1.52E-49	0.00	0.02	0.45
Fe	rs855791	*TMPRSS6*	G	A	0.06	0.007	5.02E-15	0.00	0.02	0.40
Fe	rs9990333	*TFRC*	C	T	0.05	0.007	1E-13	0.02	0.02	0.18
Cu	rs1175550	*SMIM1*	G	A	0.20	0.032	2.51E-10	-0.05	0.02	0.01
Cu	rs2769264	*SELENBP1*	G	T	0.31	0.034	1.49E-20	-0.03	0.02	0.08
Zn	rs1532423	*CA1*	A	G	0.18	0.026	2.98E-12	-0.02	0.02	0.10
Zn	rs2120019	*PPCDC*	T	C	0.29	0.033	9.94E-19	0.01	0.02	0.26

Supplementary Figure 7. MR study of Mg and BD.

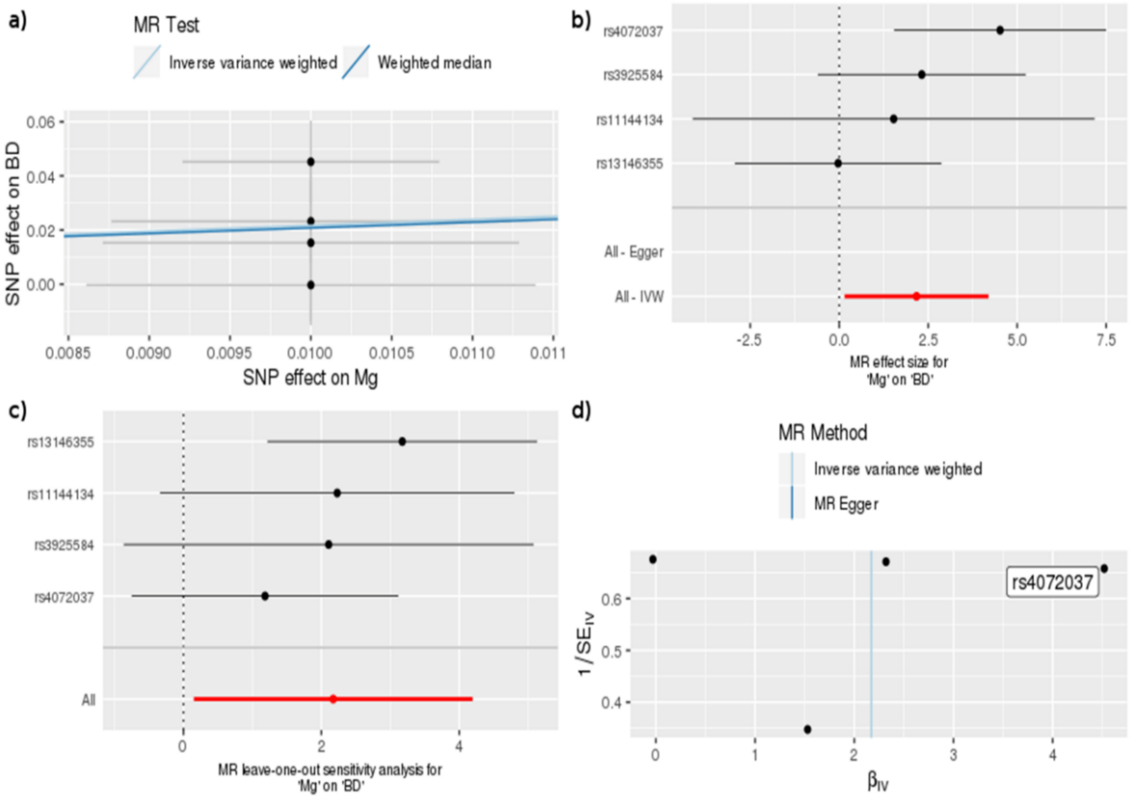

Supplementary Figure 8. MR study of Cu and BD.

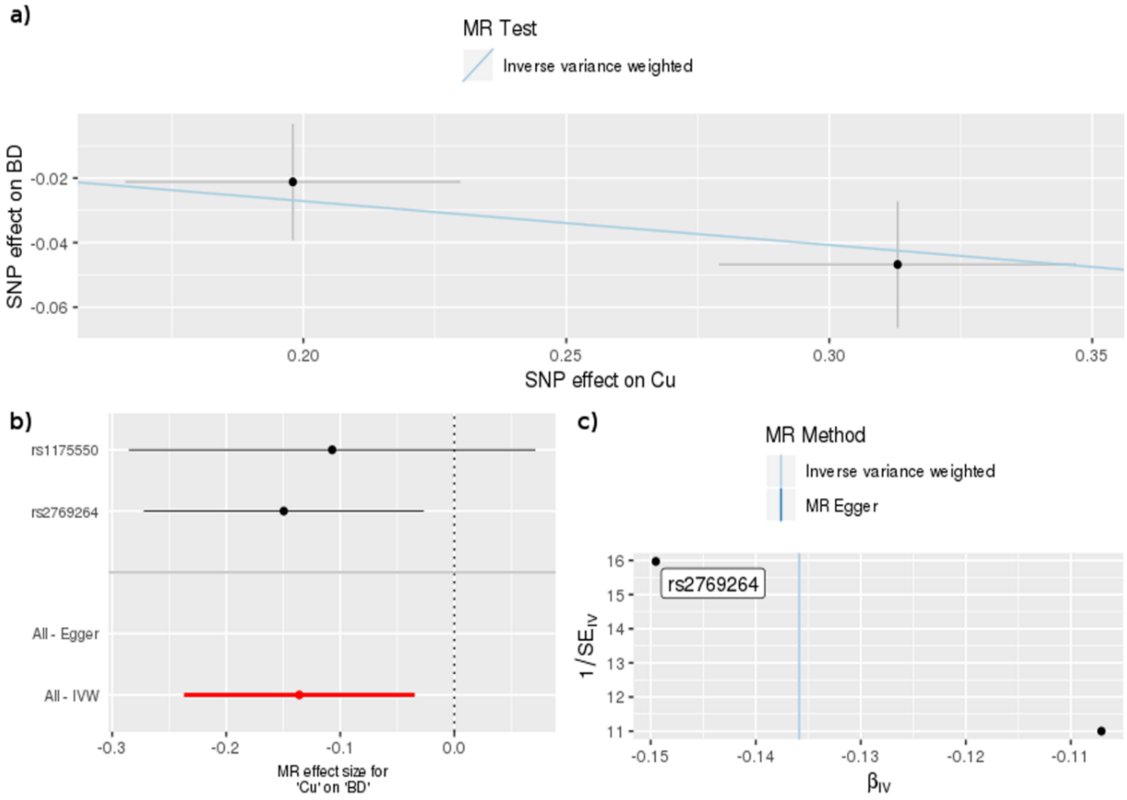

Supplementary Table 7. Summary statistics for the genetic variants associated with the exposure factors investigated for an association with BD in the present Mendelian randomization study.

Exp	SNP	Gene	Effect_ allele	Other_ allele	Beta_ exp	Se_ exp	Pval_ exp	Beta_ out	Se_ out	Pval_ out
Ca	rs10491003	*LINC00709*	T	C	0.027	0.005	2.51E-09	-0.034	0.026	0.093
Ca	rs1550532	*DGKD*	C	G	0.018	0.003	2.97E-11	-0.010	0.016	0.267
Ca	rs1570669	*CYP24A1*	G	A	0.018	0.003	4.53E-12	-0.008	0.016	0.314
Ca	rs17711722	*GTF2IP5*	T	C	0.015	0.003	3.98E-09	0.043	0.017	0.005
Ca	rs1801725	*CASR*	T	G	0.071	0.004	4.33E-86	0.039	0.021	0.032
Ca	rs7336933	*VWA8-AS1*	G	A	0.022	0.004	4.49E-10	0.029	0.020	0.081
Ca	rs7481584	*CARS*	G	A	0.018	0.003	4.97E-11	-0.020	0.016	0.109
Ca	rs780094	*GCKR*	T	C	0.017	0.003	5.02E-11	0.058	0.015	0.000
Mg	rs11144134	*TRPM6*	C	T	0.01	0.001	3.92E-15	0.015	0.029	0.298
Mg	rs13146355	*SHROOM3*	A	G	0.01	0.001	3.02E-13	0.000	0.015	0.492
Mg	rs3925584	*DCDC1*	T	C	0.01	0.001	2.52E-16	0.023	0.015	0.060
Mg	rs4072037	*MUC1*	T	C	0.01	0.001	1.00E-36	0.045	0.015	0.001
Fe	rs174577	*FADS2*	A	C	0.062	0.007	9.96E-18	0.071	0.016	0.000
Fe	rs1800562	*HFE*	G	A	0.479	0.016	5.21E-196	0.053	0.031	0.046
Fe	rs411988	*TEX14*	G	A	0.044	0.007	1.00E-10	-0.005	0.015	0.375
Fe	rs4921915	*NAT2*	A	G	0.079	0.009	3.52E-19	0.017	0.018	0.169
Fe	rs6486121	*ARNTL*	C	T	0.046	0.007	2.00E-10	-0.052	0.015	0.000
Fe	rs651007	*ABO*	C	T	0.05	0.009	5.00E-09	0.034	0.018	0.027
Fe	rs7385804	*TFR2*	A	C	0.064	0.007	5.01E-19	-0.009	0.015	0.273
Fe	rs744653	*AC013439.4*	C	T	0.089	0.01	4.16E-19	0.010	0.021	0.310
Fe	rs8177179	*TF*	G	A	0.154	0.01	1.52E-49	-0.019	0.015	0.102
Fe	rs855791	*TMPRSS6*	G	A	0.055	0.007	5.02E-15	0.013	0.015	0.198
Fe	rs9990333	*TFRC*	C	T	0.051	0.007	1.00E-13	0.003	0.015	0.424
Cu	rs1175550	*SMIM1*	G	A	0.198	0.032	2.51E-10	-0.021	0.018	0.119
Cu	rs2769264	*SELENBP1*	G	T	0.313	0.034	1.49E-20	-0.047	0.020	0.008
Zn	rs1532423	*CA1*	A	G	0.178	0.026	2.98E-12	0.017	0.015	0.126
Zn	rs2120019	*PPCDC*	T	C	0.287	0.033	9.94E-19	-0.006	0.018	0.372

Supplementary Table 8. Summary statistics for the genetic variants associated with the exposure factors investigated for an association with SCZ in the present Mendelian randomization study.

Exp	SNP	Gene	Effect_ allele	Other_ allele	Beta_ exp	Se_ exp	Pval_ exp	Beta_ out	Se_ out	Pval_ out
Ca	rs10491003	*LINC00709*	T	C	0.027	0.005	2.51E-09	0.010	0.021	0.314
Ca	rs1550532	*DGKD*	C	G	0.018	0.003	2.97E-11	-0.004	0.013	0.369
Ca	rs1570669	*CYP24A1*	G	A	0.018	0.003	4.53E-12	-0.005	0.013	0.356
Ca	rs17711722	*GTF2IP5*	T	C	0.015	0.003	3.98E-09	-0.003	0.014	0.426
Ca	rs1801725	*CASR*	T	G	0.071	0.004	4.33E-86	-0.004	0.017	0.413
Ca	rs7336933	*VWA8-AS1*	G	A	0.022	0.004	4.49E-10	-0.044	0.017	0.004
Ca	rs7481584	*CARS*	G	A	0.018	0.003	4.97E-11	-0.015	0.013	0.128
Ca	rs780094	*GCKR*	T	C	0.017	0.003	5.02E-11	0.002	0.012	0.435
Mg	rs11144134	*TRPM6*	C	T	0.010	0.001	3.92E-15	-0.008	0.023	0.364
Mg	rs13146355	*SHROOM3*	A	G	0.010	0.001	3.02E-13	-0.001	0.012	0.480
Mg	rs3925584	*DCDC1*	T	C	0.010	0.001	2.52E-16	-0.013	0.012	0.132
Mg	rs4072037	*MUC1*	T	C	0.010	0.001	1.00E-36	0.012	0.012	0.161
Fe	rs174577	*FADS2*	A	C	0.062	0.007	9.96E-18	0.017	0.013	0.087
Fe	rs411988	*TEX14*	G	A	0.044	0.007	1.00E-10	0.005	0.012	0.324
Fe	rs4921915	*NAT2*	A	G	0.079	0.009	3.52E-19	-0.003	0.014	0.431
Fe	rs6486121	*ARNTL*	C	T	0.046	0.007	2.00E-10	0.013	0.012	0.142
Fe	rs651007	*ABO*	C	T	0.050	0.009	5.00E-09	-0.002	0.015	0.445
Fe	rs7385804	*TFR2*	A	C	0.064	0.007	5.01E-19	-0.027	0.013	0.016
Fe	rs744653	*AC013439.4*	C	T	0.089	0.010	4.16E-19	0.030	0.017	0.042
Fe	rs8177179	*TF*	G	A	0.154	0.010	1.52E-49	-0.004	0.012	0.360
Fe	rs855791	*TMPRSS6*	G	A	0.055	0.007	5.02E-15	0.014	0.012	0.121
Fe	rs9990333	*TFRC*	C	T	0.051	0.007	1.00E-13	0.010	0.012	0.195
Cu	rs1175550	*SMIM1*	G	A	0.198	0.032	2.51E-10	0.009	0.014	0.271
Cu	rs2769264	*SELENBP1*	G	T	0.313	0.034	1.49E-20	-0.027	0.016	0.041
Zn	rs1532423	*CA1*	A	G	0.178	0.026	2.98E-12	-0.002	0.012	0.438
Zn	rs2120019	*PPCDC*	T	C	0.287	0.033	9.94E-19	-0.028	0.015	0.030

Supplementary Table 9. Summary statistics for the genetic variants associated with the exposure factors investigated for an association with PD in the present Mendelian randomization study.

Exp	SNP	Gene	Effect_ allele	Other_ allele	Beta_ exp	Se_ exp	Pval_ exp	Beta_ out	Se_ out	Pval_ out
Ca	rs10491003	*LINC00709*	T	C	0.027	0.005	2.51E-09	-0.043	0.057	0.225
Ca	rs1550532	*DGKD*	C	G	0.018	0.003	2.97E-11	0.041	0.035	0.123
Ca	rs1570669	*CYP24A1*	G	A	0.018	0.003	4.53E-12	-0.030	0.034	0.188
Ca	rs17711722	*GTF2IP5*	T	C	0.015	0.003	3.98E-09	0.071	0.033	0.015
Ca	rs1801725	*CASR*	T	G	0.071	0.004	4.33E-86	0.043	0.046	0.178
Ca	rs7336933	*VWA8-AS1*	G	A	0.022	0.004	4.49E-10	-0.035	0.046	0.222
Ca	rs7481584	*CARS*	G	A	0.018	0.003	4.97E-11	0.043	0.037	0.120
Ca	rs780094	*GCKR*	T	C	0.017	0.003	5.02E-11	-0.004	0.033	0.456
Mg	rs11144134	*TRPM6*	C	T	0.01	0.001	3.92E-15	-0.048	0.064	0.226
Mg	rs13146355	*SHROOM3*	A	G	0.01	0.001	3.02E-13	0.025	0.033	0.224
Mg	rs3925584	*DCDC1*	T	C	0.01	0.001	2.52E-16	-0.011	0.033	0.376
Mg	rs4072037	*MUC1*	T	C	0.01	0.001	1.00E-36	-0.010	0.032	0.381
Mg	rs7965584	*RP11-654D12.2*	A	G	0.07	0.008	5.01E-17	-0.005	0.036	0.443
Fe	rs174577	*FADS2*	A	C	0.062	0.007	9.96E-18	-0.019	0.035	0.288
Fe	rs1800562	*HFE*	G	A	0.479	0.016	5.21E-196	0.037	0.068	0.293
Fe	rs411988	*TEX14*	G	A	0.044	0.007	1.00E-10	0.039	0.033	0.118
Fe	rs4921915	*NAT2*	A	G	0.079	0.009	3.52E-19	-0.019	0.039	0.309
Fe	rs6486121	*ARNTL*	C	T	0.046	0.007	2.00E-10	0.055	0.034	0.052
Fe	rs651007	*ABO*	C	T	0.05	0.009	5.00E-09	-0.063	0.040	0.056
Fe	rs7385804	*TFR2*	A	C	0.064	0.007	5.01E-19	0.003	0.034	0.462
Fe	rs744653	*AC013439.4*	C	T	0.089	0.01	4.16E-19	-0.058	0.047	0.107
Fe	rs8177179	*TF*	G	A	0.154	0.01	1.52E-49	-0.036	0.033	0.133
Fe	rs855791	*TMPRSS6*	G	A	0.055	0.007	5.02E-15	-0.007	0.033	0.417
Fe	rs9990333	*TFRC*	C	T	0.051	0.007	1.00E-13	-0.014	0.033	0.337
Cu	rs1175550	*SMIM1*	G	A	0.198	0.032	2.51E-10	0.045	0.040	0.128
Cu	rs2769264	*SELENBP1*	G	T	0.313	0.034	1.49E-20	0.023	0.043	0.295
Zn	rs1532423	*CA1*	A	G	0.178	0.026	2.98E-12	-0.046	0.033	0.080
Zn	rs2120019	*PPCDC*	T	C	0.287	0.033	9.94E-19	0.003	0.040	0.468

Supplementary Table 10. Summary statistics for the genetic variants associated with the exposure factors investigated for an association with MDD in the present Mendelian randomization study.

Exp	SNP	Gene	Effect_ allele	Other_ allele	Beta_ exp	Se_ exp	Pval_ exp	Beta_ out	Se_ out	Pval_ out
Ca	rs10491003	*LINC00709*	T	C	0.027	0.005	2.51E-09	0.033	0.030	0.138
Ca	rs1550532	*DGKD*	C	G	0.018	0.003	2.97E-11	-0.003	0.008	0.366
Ca	rs1570669	*CYP24A1*	G	A	0.018	0.003	4.53E-12	0.000	0.007	0.492
Ca	rs17711722	*GTF2IP5*	T	C	0.015	0.003	3.98E-09	0.001	0.009	0.451
Ca	rs1801725	*CASR*	T	G	0.071	0.004	4.33E-86	0.002	0.020	0.451
Ca	rs7481584	*CARS*	G	A	0.018	0.003	4.97E-11	-0.002	0.007	0.403
Ca	rs780094	*GCKR*	T	C	0.017	0.003	5.02E-11	-0.011	0.007	0.057
Mg	rs13146355	*SHROOM3*	A	G	0.01	0.001	3.02E-13	0.013	0.008	0.054
Mg	rs3925584	*DCDC1*	T	C	0.01	0.001	2.52E-16	-0.013	0.007	0.039
Mg	rs4072037	*MUC1*	T	C	0.01	0.001	1.00E-36	0.011	0.009	0.112
Fe	rs174577	*FADS2*	A	C	0.062	0.007	9.96E-18	0.003	0.007	0.311
Fe	rs411988	*TEX14*	G	A	0.044	0.007	1.00E-10	-0.005	0.009	0.270
Fe	rs4921915	*NAT2*	A	G	0.079	0.009	3.52E-19	0.001	0.007	0.422
Fe	rs6486121	*ARNTL*	C	T	0.046	0.007	2.00E-10	-0.006	0.009	0.278
Fe	rs651007	*ABO*	C	T	0.05	0.009	5.00E-09	-0.003	0.008	0.336
Fe	rs7385804	*TFR2*	A	C	0.064	0.007	5.01E-19	0.001	0.009	0.432
Fe	rs744653	*AC013439.4*	C	T	0.089	0.01	4.16E-19	-0.011	0.034	0.377
Fe	rs8177179	*TF*	G	A	0.154	0.01	1.52E-49	-0.005	0.008	0.261
Fe	rs9990333	*TFRC*	C	T	0.051	0.007	1.00E-13	-0.012	0.034	0.362
Cu	rs2769264	*SELENBP1*	G	T	0.313	0.034	1.49E-20	0.010	0.008	0.103
Zn	rs1532423	*CA1*	A	G	0.178	0.026	2.98E-12	-0.001	0.007	0.429
Zn	rs2120019	*PPCDC*	T	C	0.287	0.033	9.94E-19	-0.003	0.007	0.341

Gene abbreviation:

LINC00709, long intergenic non-protein coding RNA 709;
DGKD, diacylglycerol kinase delta;
CYP24A1, cytochrome P450 family 24 subfamily A member 1;
GTF2IP5, general transcription factor IIi pseudogene 5;
CASR, calcium sensing receptor;
VWA8-AS1, VWA8 antisense RNA 1 (head to head);
CARS, cysteinyl-tRNA synthetase;
GCKR, glucokinase regulator;
TRPM6, transient receptor potential cation channel subfamily M member 6,
SHROOM3, shroom family member 3;
DCDC1, doublecortin domain containing 1;
MUC1, mucin 1, cell surface associated;
RP11 (PRPF31), pre-mRNA processing factor 31,
FADS2, fatty acid desaturase 2;
HFE, homeostatic iron regulator;
TEX14, testis expressed 14, intercellular bridge forming factor;
NAT2, N-acetyltransferase 2;
ARNTL, aryl hydrocarbon receptor nuclear translocator like;
ABO, alpha 1-3-N-acetylgalactosaminyltransferase and alpha 1-3-galactosyltransferase;
TFR2, transferrin receptor 2;
AC013439.4, not available;
TF, transmembrane serine protease 6;
TMPRSS6, transmembrane serine protease 6;
TFRC, transferrin receptor;
SMIM1, small integral membrane protein 1 (Vel blood group);
SELENBP1, selenium binding protein 1;
CA1, carbonic anhydrase 1;
PPCDC, phosphopantothenoylcysteine decarboxylase.

References

1. Bristow, S.M.; Gamble, G.D.; Horne, A.M.; Reid, I.R. Dietary calcium intake and rate of bone loss in men. *The British journal of nutrition* **2017**, *117*, 1432-1438, doi:10.1017/S0007114517001301.
2. Welch, A.A.; Skinner, J.; Hickson, M. Dietary Magnesium May Be Protective for Aging of Bone and Skeletal Muscle in Middle and Younger Older Age Men and Women: Cross-Sectional Findings from the UK Biobank Cohort. *Nutrients* **2017**, *9*, doi:10.3390/nu9111189.
3. Kunutsor, S.K.; Whitehouse, M.R.; Blom, A.W.; Laukkanen, J.A. Low serum magnesium levels are associated with increased risk of fractures: a long-term prospective cohort study. *European journal of epidemiology* **2017**, *32*, 593-603, doi:10.1007/s10654-017-0242-2.
4. Finck, H.; Hart, A.; Lentjes, M.; Jennings, A.; Luben, R.; Khaw, K.T.; Welch, A. Prospective associations between dietary iron intake and serum ferritin concentrations with fracture risk in EPIC-Norfolk men and women. *Proceedings of the Nutrition Society* **2015**, *74*.
5. Kim, D.E.; Cho, S.H.; Park, H.M.; Chang, Y.K. Relationship between bone mineral density and dietary intake of beta-carotene, vitamin C, zinc and vegetables in postmenopausal Korean women: a cross-sectional study. *The Journal of international medical research* **2016**, *44*, 1103-1114, doi:10.1177/0300060516662402.
6. Qu, X.; He, Z.; Qiao, H.; Zhai, Z.; Mao, Z.; Yu, Z.; Dai, K. Serum copper levels are associated with bone mineral density and total fracture. *Journal of orthopaedic translation* **2018**, *14*, 34-44, doi:10.1016/j.jot.2018.05.001.
7. Liu, J.H.; Ng, M.Y.; Cheung, T.; Chung, H.Y.; Chen, Y.; Zhen, Z.; Zou, Y.; Mak, K.F.; Khong, P.L.; Lau, C.S., et al. Ten-year progression of coronary artery, carotid artery, and aortic calcification in patients with rheumatoid arthritis. *Clinical rheumatology* **2017**, *36*, 807-816, doi:10.1007/s10067-016-3536-y.
8. dos Santos, A.T.; Assuncao, A.A.Q.; Foschetti, D.A.; Uchoa, F.N.M.; Alves, N.; Aragao, K.S. Assessment of nutritional and biochemical status in patients with rheumatoid arthritis undergoing pharmacological treatment. A pilot study. *International Journal of Clinical and Experimental Medicine* **2016**, *9*, 4282-4290.
9. Benito-Garcia, E.; Feskanich, D.; Hu, F.B.; Mandl, L.A.; Karlson, E.W. Protein, iron, and meat consumption and risk for rheumatoid arthritis: a prospective cohort study. *Arthritis Research & Therapy* **2007**, *9*, R16-R16.
10. Cerhan, J.R.; Saag, K.G.; Merlino, L.A.; Mikuls, T.R.; Criswell, L.A. Antioxidant micronutrients and risk of rheumatoid arthritis in a cohort of older women. *American journal of epidemiology* **2003**, *157*, 345-354.
11. Squitti, R.; Mendez, A.J.; Simonelli, I.; Ricordi, C. Diabetes and Alzheimer's Disease: Can Elevated Free Copper Predict the Risk of the Disease? *Journal of Alzheimer's disease : JAD* **2017**, *56*, 1055-1064, doi:10.3233/JAD-161033.
12. Fang, X.; Han, H.; Li, M.; Liang, C.; Fan, Z.; Aaseth, J.; He, J.; Montgomery, S.; Cao, Y. Dose-Response Relationship between Dietary Magnesium Intake and Risk of Type 2 Diabetes Mellitus: A Systematic Review and Meta-Regression Analysis of Prospective Cohort Studies. *Nutrients* **2016**, *8*, doi:10.3390/nu8110739.
13. Kim, K.N.; Oh, S.Y.; Hong, Y.C. Associations of serum calcium levels and dietary calcium intake with incident type 2 diabetes over 10 years: the Korean Genome and Epidemiology Study (KoGES). *Diabetology & metabolic syndrome* **2018**, *10*, 50, doi:10.1186/s13098-018-0349-

y.
14. Villegas, R.; Gao, Y.T.; Dai, Q.; Yang, G.; Cai, H.; Li, H.; Zheng, W.; Shu, X.O. Dietary calcium and magnesium intakes and the risk of type 2 diabetes: the Shanghai Women's Health Study. *The American journal of clinical nutrition* **2009**, *89*, 1059-1067, doi:10.3945/ajcn.2008.27182.
15. Drake, I.; Hindy, G.; Ericson, U.; Orho-Melander, M. A prospective study of dietary and supplemental zinc intake and risk of type 2 diabetes depending on genetic variation in SLC30A8. *Genes & nutrition* **2017**, *12*, 30, doi:10.1186/s12263-017-0586-y.
16. Podmore, C.; Meidtner, K.; Schulze, M.B.; Scott, R.A.; Ramond, A.; Butterworth, A.S.; Di Angelantonio, E.; Danesh, J.; Arriola, L.; Barricarte, A., et al. Association of Multiple Biomarkers of Iron Metabolism and Type 2 Diabetes: The EPIC-InterAct Study. *Diabetes care* **2016**, *39*, 572-581, doi:10.2337/dc15-0257.
17. Zhao, Z.; Li, S.; Liu, G.; Yan, F.; Ma, X.; Huang, Z.; Tian, H. Body iron stores and heme-iron intake in relation to risk of type 2 diabetes: a systematic review and meta-analysis. *PloS one* **2012**, *7*, e41641, doi:10.1371/journal.pone.0041641.
18. Cherbuin, N.; Kumar, R.; Sachdev, P.S.; Anstey, K.J. Dietary Mineral Intake and Risk of Mild Cognitive Impairment: The PATH through Life Project. *Frontiers in aging neuroscience* **2014**, *6*, 4, doi:10.3389/fnagi.2014.00004.
19. Ozawa, M.; Ninomiya, T.; Ohara, T.; Hirakawa, Y.; Doi, Y.; Hata, J.; Uchida, K.; Shirota, T.; Kitazono, T.; Kiyohara, Y. Self-reported dietary intake of potassium, calcium, and magnesium and risk of dementia in the Japanese: the Hisayama Study. *Journal of the American Geriatrics Society* **2012**, *60*, 1515-1520, doi:10.1111/j.1532-5415.2012.04061.x.
20. Strozyk, D.; Launer, L.J.; Adlard, P.A.; Cherny, R.A.; Tsatsanis, A.; Volitakis, I.; Blennow, K.; Petrovitch, H.; White, L.R.; Bush, A.I. Zinc and copper modulate Alzheimer Abeta levels in human cerebrospinal fluid. *Neurobiology of aging* **2009**, *30*, 1069-1077, doi:10.1016/j.neurobiolaging.2007.10.012.
21. Rembach, A.; Hare, D.J.; Doecke, J.D.; Burnham, S.C.; Volitakis, I.; Fowler, C.J.; Cherny, R.A.; McLean, C.; Grimm, R.; Martins, R., et al. Decreased serum zinc is an effect of ageing and not Alzheimer's disease. *Metallomics : integrated biometal science* **2014**, *6*, 1216-1219, doi:10.1039/c4mt00060a.
22. Smorgon, C.; Mari, E.; Atti, A.R.; Dalla Nora, E.; Zamboni, P.F.; Calzoni, F.; Passaro, A.; Fellin, R. Trace elements and cognitive impairment: an elderly cohort study. *Archives of gerontology and geriatrics. Supplement* **2004**, 10.1016/j.archger.2004.04.050, 393-402, doi:10.1016/j.archger.2004.04.050.
23. Siwek, M.; Styczen, K.; Sowa-Kucma, M.; Dudek, D.; Reczynski, W.; Szewczyk, B.; Misztak, P.; Opoka, W.; Topor-Madry, R.; Nowak, G. The serum concentration of magnesium as a potential state marker in patients with diagnosis of bipolar disorder. *Psychiatria polska* **2015**, *49*, 1277-1287, doi:10.12740/PP/OnlineFirst/42047.
24. Siwek, M.; Styczen, K.; Sowa-Kucma, M.; Dudek, D.; Reczynski, W.; Szewczyk, B.; Misztak, P.; Opoka, W.; Topor-Madry, R.; Nowak, G., et al. The serum concentration of copper in bipolar disorder. *Psychiatria polska* **2017**, *51*, 469-481, doi:10.12740/PP/OnlineFirst/65250.
25. Styczen, K.; Sowa-Kucma, M.; Dudek, D.; Siwek, M.; Reczynski, W.; Szewczyk, B.; Misztak, P.; Topor-Madry, R.; Opoka, W.; Nowak, G. Zinc and copper concentration do not differentiate bipolar disorder from major depressive disorder. *Psychiatria polska* **2018**, *52*,

449-457, doi:10.12740/PP/OnlineFirst/80069.
26. Gonzalez-Estecha, M.; Trasobares, E.M.; Tajima, K.; Cano, S.; Fernandez, C.; Lopez, J.L.; Unzeta, B.; Arroyo, M.; Fuentenebro, F. Trace elements in bipolar disorder. *Journal of trace elements in medicine and biology : organ of the Society for Minerals and Trace Elements* **2011**, *25 Suppl 1*, S78-83, doi:10.1016/j.jtemb.2010.10.015.
27. Nielsen, P.R.; Meyer, U.; Mortensen, P.B. Individual and combined effects of maternal anemia and prenatal infection on risk for schizophrenia in offspring. *Schizophrenia research* **2016**, *172*, 35-40, doi:10.1016/j.schres.2016.02.025.
28. Vidovic, B.; Dordevic, B.; Milovanovic, S.; Skrivanj, S.; Pavlovic, Z.; Stefanovic, A.; Kotur-Stevuljevic, J. Selenium, zinc, and copper plasma levels in patients with schizophrenia: relationship with metabolic risk factors. *Biological trace element research* **2013**, *156*, 22-28, doi:10.1007/s12011-013-9842-1.
29. Miyake, Y.; Tanaka, K.; Fukushima, W.; Sasaki, S.; Kiyohara, C.; Tsuboi, Y.; Yamada, T.; Oeda, T.; Miki, T.; Kawamura, N., et al. Lack of association of dairy food, calcium, and vitamin D intake with the risk of Parkinson's disease: a case-control study in Japan. *Parkinsonism & related disorders* **2011**, *17*, 112-116, doi:10.1016/j.parkreldis.2010.11.018.
30. Zhao, H.W.; Lin, J.; Wang, X.B.; Cheng, X.; Wang, J.Y.; Hu, B.L.; Zhang, Y.; Zhang, X.; Zhu, J.H. Assessing plasma levels of selenium, copper, iron and zinc in patients of Parkinson's disease. *PloS one* **2013**, *8*, e83060, doi:10.1371/journal.pone.0083060.
31. Hong, C.T.; Huang, Y.H.; Liu, H.Y.; Chiou, H.Y.; Chan, L.; Chien, L.N. Newly Diagnosed Anemia Increases Risk of Parkinson's disease: A Population-Based Cohort Study. *Scientific reports* **2016**, *6*, 29651, doi:10.1038/srep29651.
32. Jacka, F.N.; Simon, O.; Robert, S.; Tell, G.S.; Ingvar, B.; Arnstein, M. Association between magnesium intake and depression and anxiety in community-dwelling adults: the Hordaland Health Study. *Aust N Z J Psychiatry* **2009**, *43*, 45-52.
33. Mills, N.T.; Maier, R.; Whitfield, J.B.; Wright, M.J.; Colodroconde, L.; Byrne, E.M.; Scott, J.G.; Byrne, G.J.; Hansell, N.K.; Vinkhuyzen, A.A.E. Investigating the relationship between iron and depression. *Journal of Psychiatric Research* **2017**, *94*, 148-155.
34. Li, Z.; Li, B.; Song, X.; Zhang, D. Dietary zinc and iron intake and risk of depression: A meta-analysis. *Psychiatry Res* **2017**, *251*, 41-47.
35. Whitfield, D.R.; Julie, V.; Amani, A.; Tibor, H.; Clive, B.; Thomas, A.J.; O'Brien, J.T.; Dag, A.; Francis, P.T. Depression and synaptic zinc regulation in Alzheimer disease, dementia with lewy bodies, and Parkinson disease dementia. *American Journal of Geriatric Psychiatry* **2015**, *23*, 141-148.
36. Roy, A.; Evers, S.E.; Avison, W.R.; Campbell, M.K. Higher zinc intake buffers the impact of stress on depressive symptoms in pregnancy. *Nutrition research* **2010**, *30*, 695-704, doi:10.1016/j.nutres.2010.09.011.
